# Author Correction: A Cu(II)–ATP complex efficiently catalyses enantioselective Diels–Alder reactions

**DOI:** 10.1038/s41467-022-28850-3

**Published:** 2022-03-22

**Authors:** Changhao Wang, Qianqian Qi, Wenying Li, Jingshuang Dang, Min Hao, Shuting Lv, Xingchen Dong, Youkun Gu, Peizhe Wu, Wenyue Zhang, Yashao Chen, Jörg S. Hartig

**Affiliations:** 1 Key Laboratory of Applied Surface and Colloid Chemistry, Ministry of Education, School of Chemistry and Chemical Engineering, Shaanxi Normal University, Xi’an, China; 2 Department of Chemistry and Konstanz Research School Chemical Biology (KoRS-CB), University of Konstanz, Konstanz, Germany

**Keywords:** Asymmetric catalysis, Catalytic mechanisms, Small molecules, Origin of life, Stereochemistry

Correction to: *Nature Communications* 10.1038/s41467-020-18554-x, published online 22 September 2020.

The original version of this Article contained several errors with regards to the absolute configurations of 2’-OH and 3’-OH at ATP in the theoretical models. The errors can be found in Fig. 4a and Fig. 4b. The correct version of Fig. 4a and 4b is:

which replaces the previous incorrect version:

This has been corrected in both the PDF and HTML versions of the Article.

The associated Supplementary Figs. S22–S25 also contained the same error. The correct version of Supplementary Figures S22-S25 are:


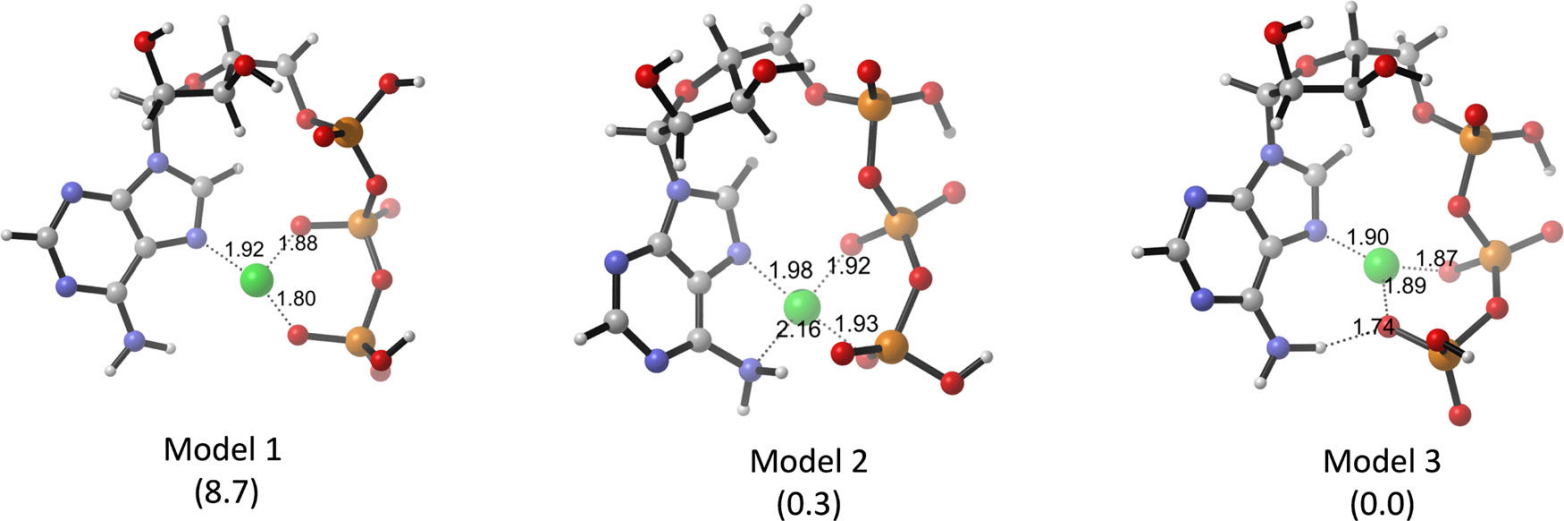


**Supplementary Fig. 22.** The proposed models of Cu^2+^⋅ATP. The relative electronic energies are in the parenthesis with a unit of kcalmol^−1^.


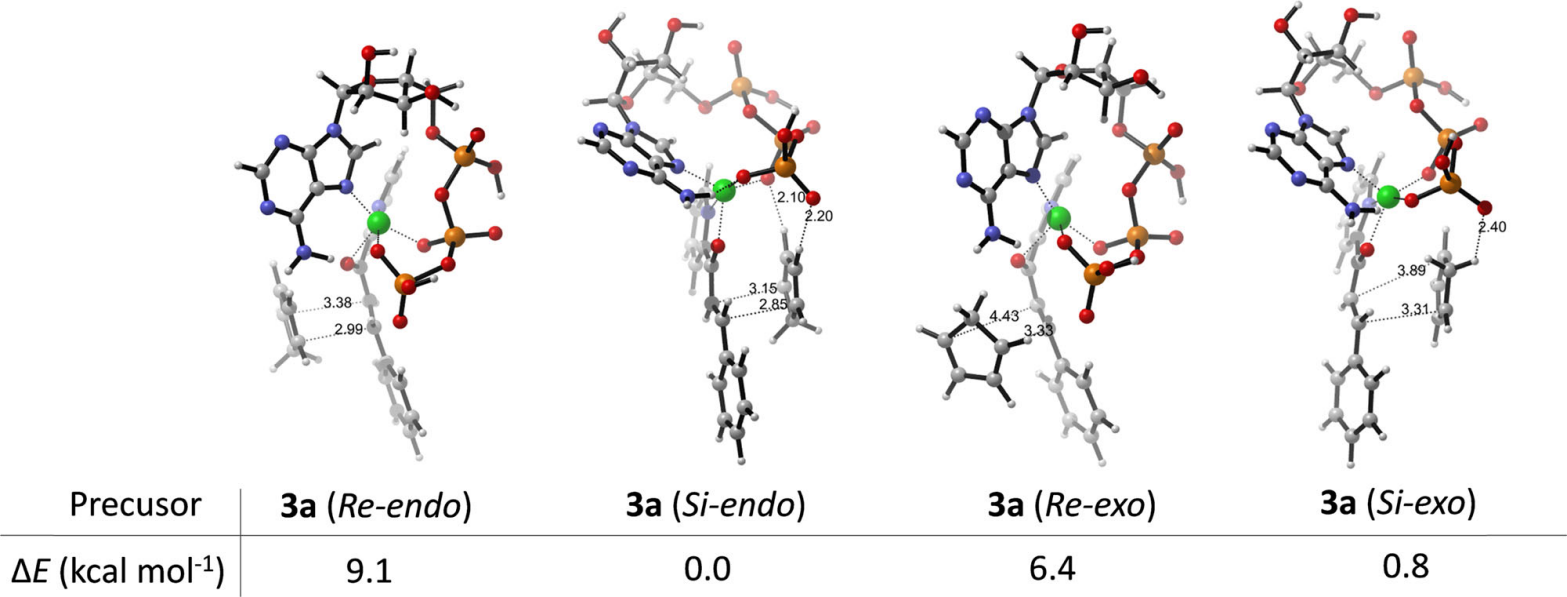


**Supplementary Fig. 23.** The precursors of the intermediates of **1a**-Cu^2+^⋅ATP and **2** that yield the corresponding products **3a** in different configurations. The relative electronic energies (ΔE) of the precursors are shown in the table.


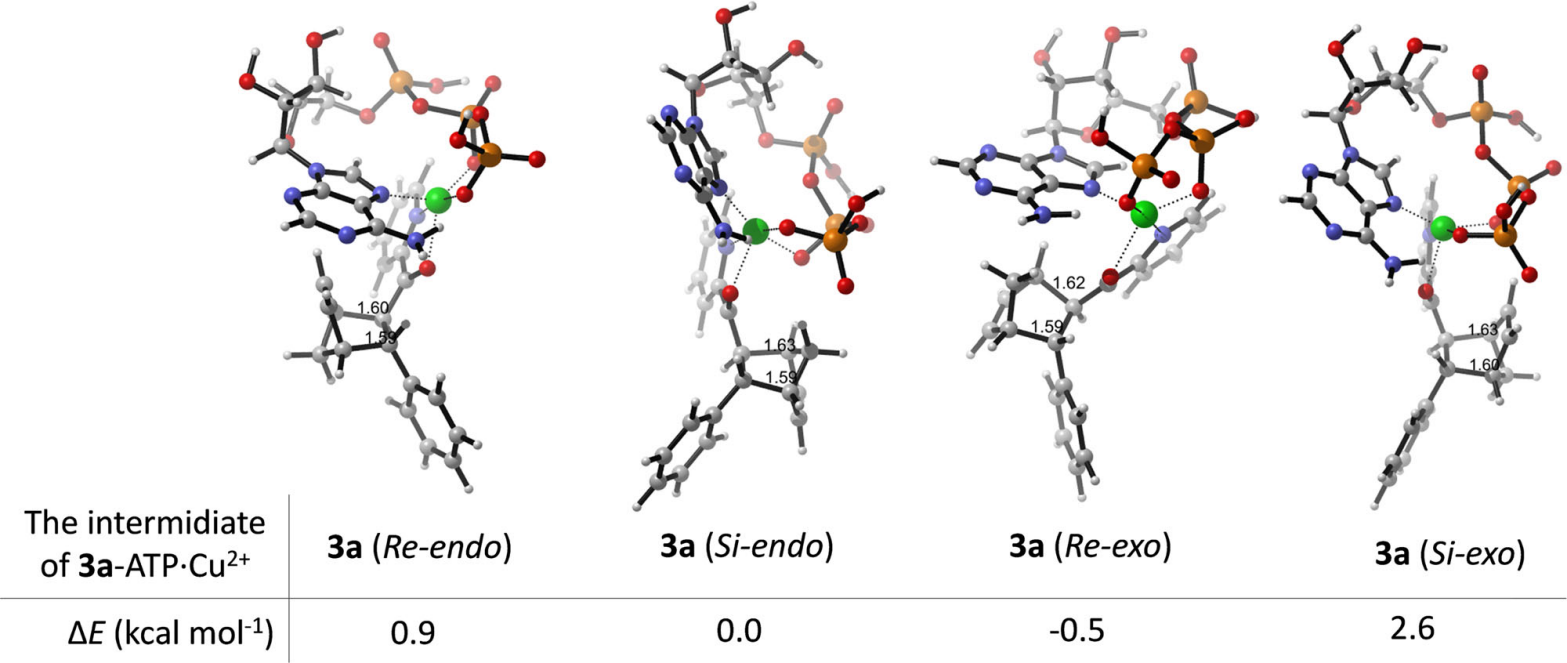


**Supplementary Fig. 24.** The intermediates of **3a**-ATP⋅Cu^2+^ and their relative electronic energies (ΔE).


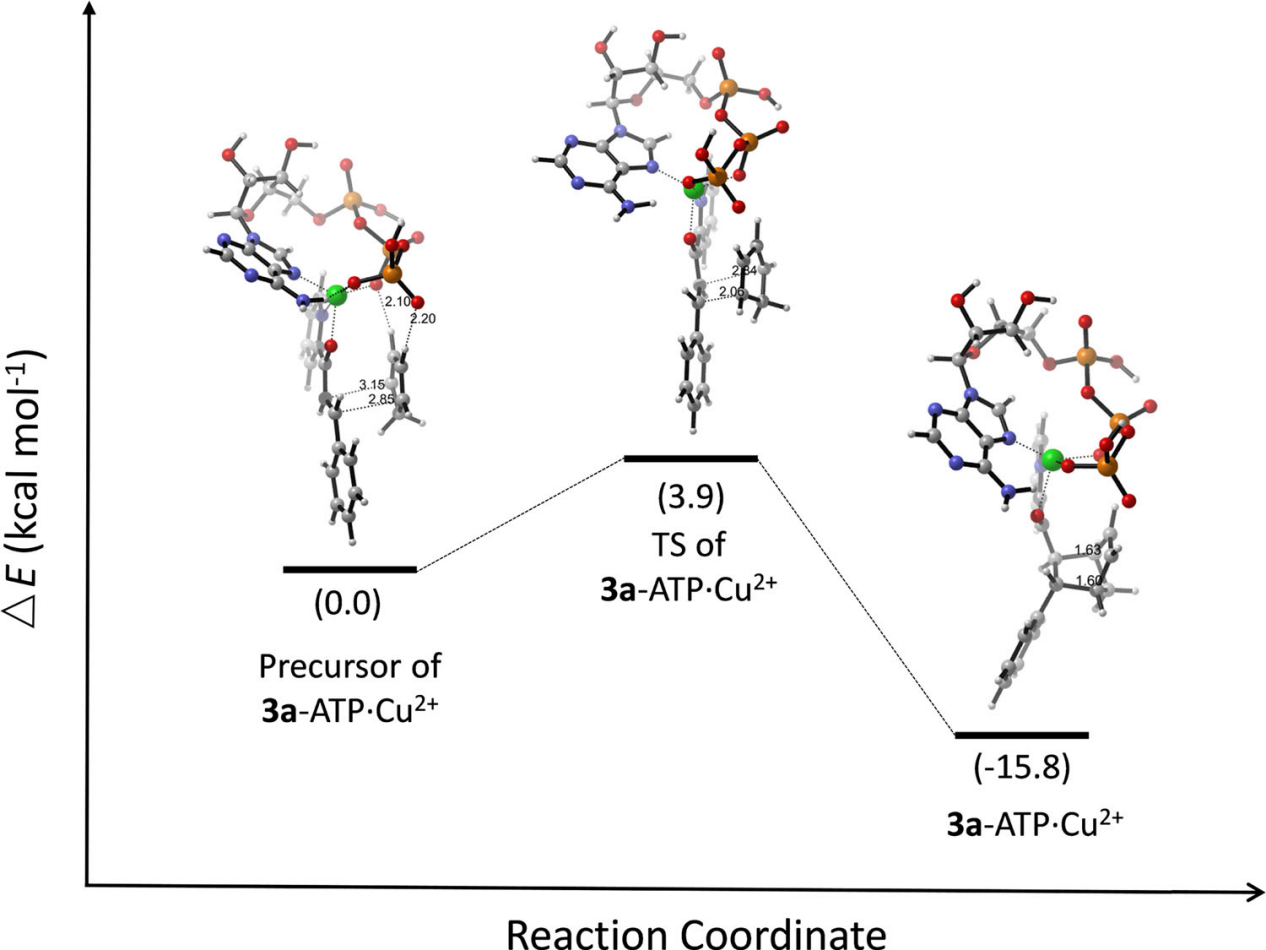


**Supplementary Fig. 25.** The relative electronic energy profile of the reaction path for Cu^2+^⋅ATP-catalyzed Diels–Alder reaction of **1a** and **2** that yields **3a** (endo) in the absolute configuration of 1R, 2S, 3S, 4S. TS, transition state.

which replace the previous incorrect versions:


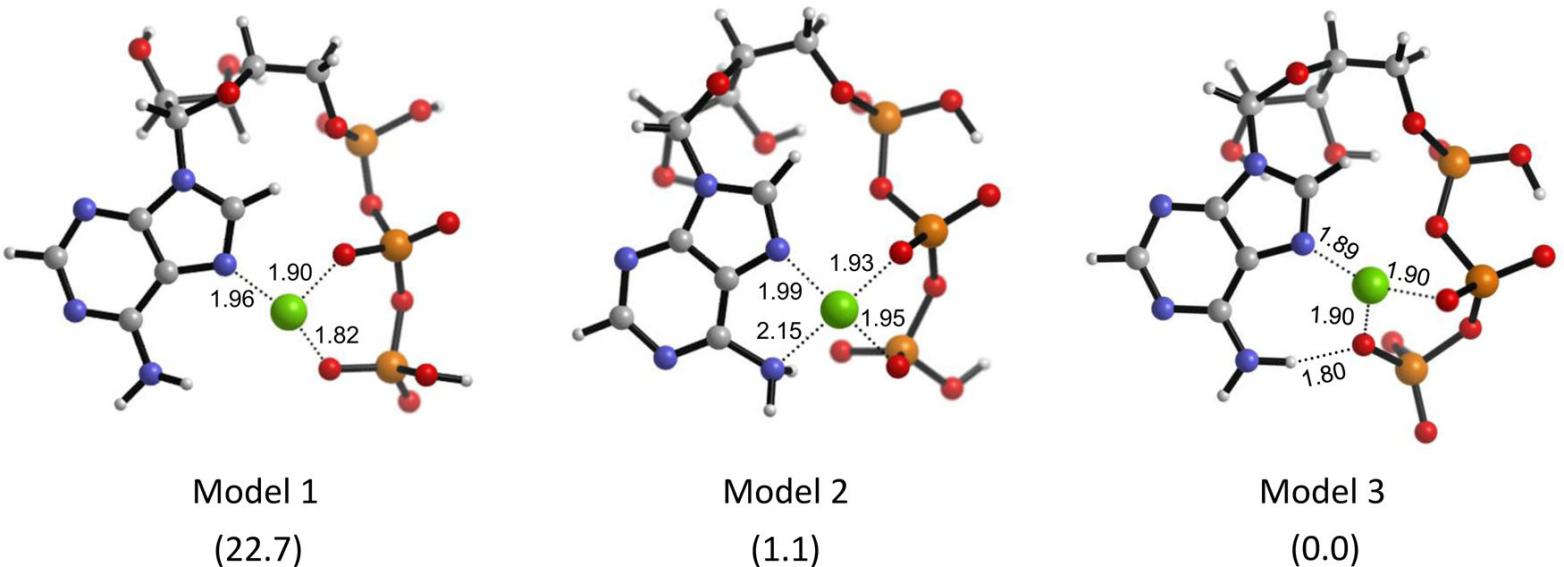


**Supplementary Fig. 22.** The proposed models of Cu^2+^⋅ATP. The relative electronic energies are in the parenthesis with a unit of kcalmol^−1^.


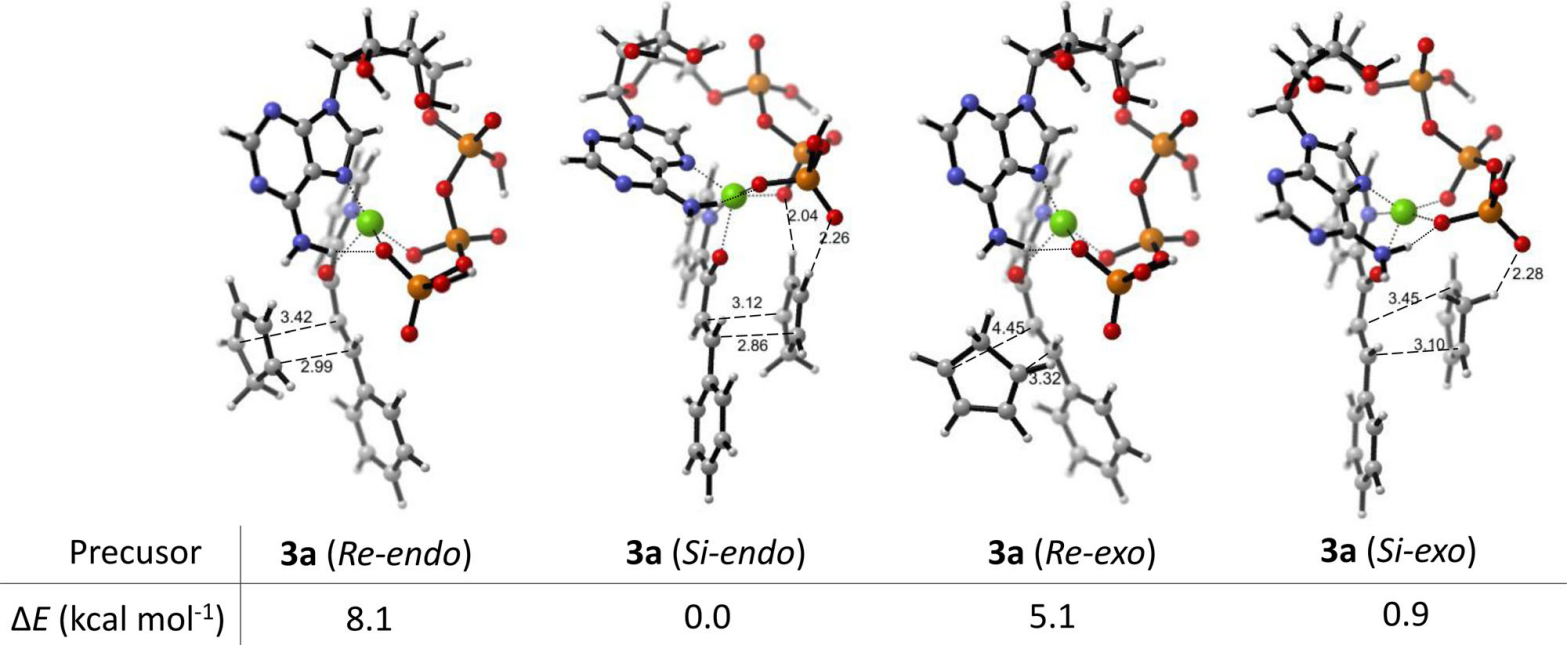


**Supplementary Fig. 23.** The precursors of the intermediates of **1a**-Cu^2+^⋅ATP and **2** that yield the corresponding products **3a** in different configurations. The relative electronic energies (ΔE) of the precursors are shown in the table.


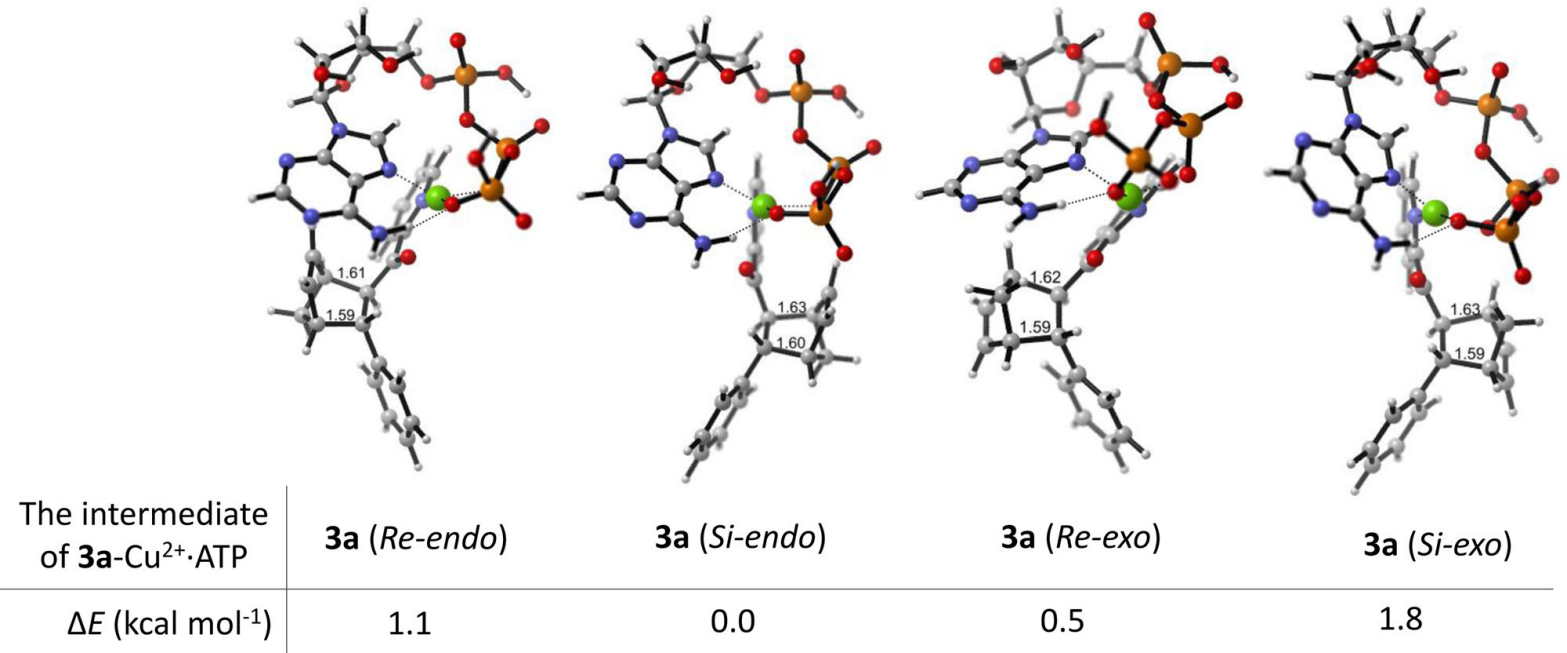


**Supplementary Fig. 24.** The intermediates of **3a**-ATP⋅Cu^2+^ and their relative electronic energies (ΔE).


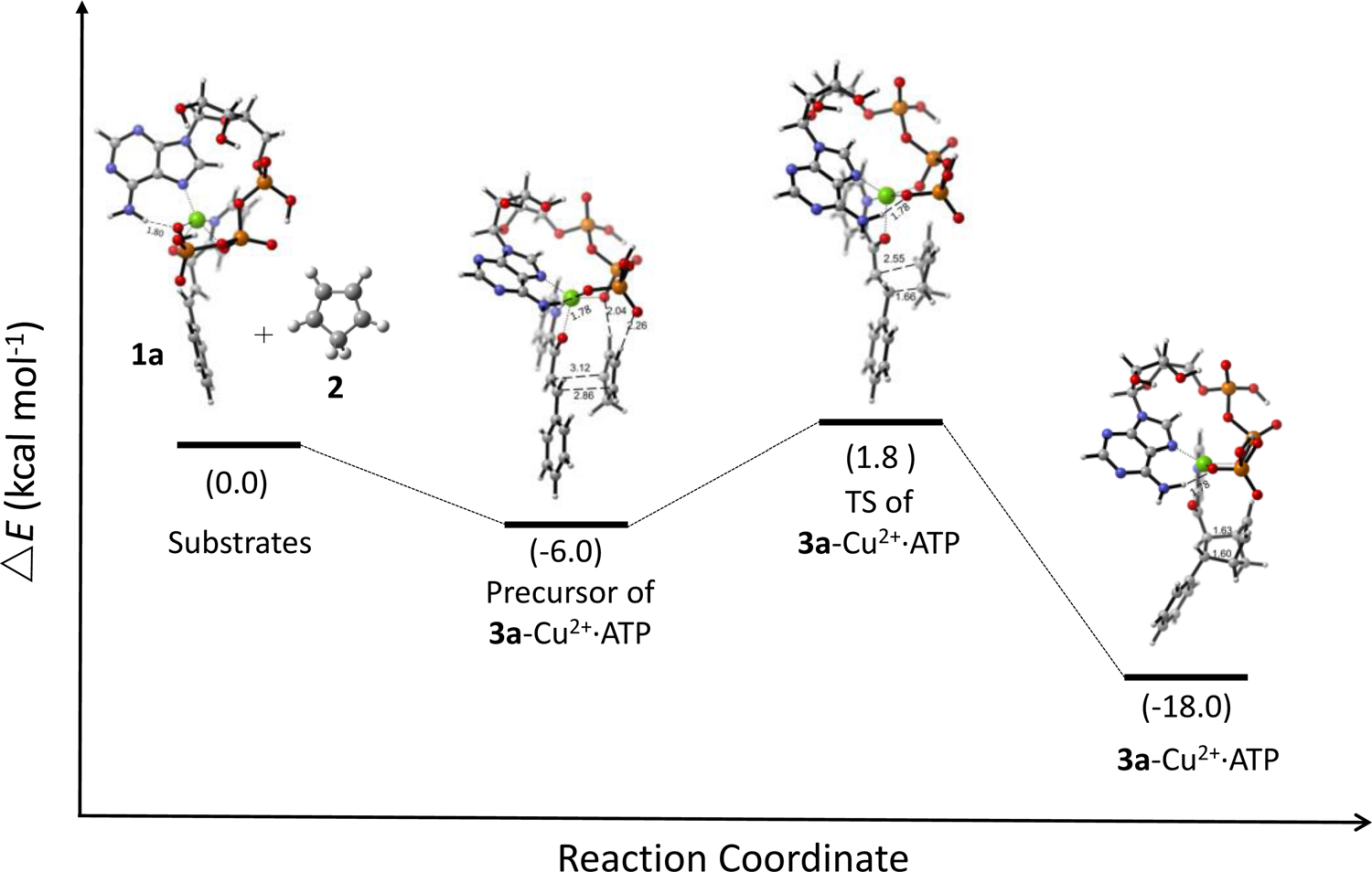


**Supplementary Fig. 25.** The relative electronic energy profile of the reaction path for Cu^2+^⋅ATP-catalyzed Diels–Alder reaction of **1a** and **2** that yields **3a** (endo) in the absolute configuration of 1R, 2S, 3S, 4S. TS, transition state.

The HTML has been updated to include a corrected version of the Supplementary Information.

Due to the above errors, the text in the original version of this Article also contained errors. Page 5, right column incorrectly reads ‘The relative electronic energy (ΔE) of the optimised Cu^2+·^ATP structure was 1.1 kcal mol^−1^ lower than that of a previously described model obtained by a molecular orbital method^57^ and 22.7 kcal mol^−1^ lower than that of the Cu^2+^·ATP model without a hydrogen bond (Supplementary Fig. S22).’ and ‘the ΔE value of the precursor of **1a**-Cu^2+^·ATP and **2** that yielded **3a** (endo) via the attack of the Si face was 8.1 kcal mol^−1^ lower than that of the precursor for the Re face attack (Fig. 4b).’ The correct version states ‘The relative electronic energy (Δ*E*) of the optimised Cu^2+^·ATP structure was 0.3 kcal mol^−1^ lower than that of a previously described model obtained by a molecular orbital method^57^ and 8.7 kcal mol^−1^ lower than that of the Cu^2+^·ATP model without a hydrogen bond (Supplementary Fig. S22).’ and ‘the Δ*E* value of the precursor of **1a**-Cu^2+^·ATP and **2** that yielded **3a** (*endo*) via the attack of the *Si* face was 9.1 kcal mol^−1^ lower than that of the precursor for the *Re* face attack (Fig. 4b).’

Page 6, left column incorrectly reads ‘However, the ΔE value of **3a** (Re-exo) was 1.3 kcal mol^−1^ lower than that of **3a** (Si-exo), in accordance with the experimental results (Supplementary Figs. S21, S24).’ The correct version states ‘However, the Δ*E* value of **3a** (*Re-exo*) was 2.6 kcal mol^−1^ lower than that of **3a** (*Si-exo*), in accordance with the experimental results (Supplementary Figs. S21, S24).’

This has been corrected in the PDF and HTML versions of the Article.

## Supplementary information

Updated Supplementary Information

