# Supplementary material for: A Cu(II)–ATP complex efficiently catalyses enantioselective Diels–Alder reactions
Source: Nat Commun. 2020 Sep 22;11:4792. doi: 10.1038/s41467-020-18554-x (PMC7508818; doi:10.1038/s41467-020-18554-x)
Supplement: Supplementary file 1 — Supplementary Information [file 41467_2020_18554_MOESM1_ESM.pdf]

Supplementary Information

A Cu(II)-ATP complex efficiently catalyses enantioselective Diels-Alder reactions

Wang *et al.*

Supplementary Tables

Supplementary Table 1 Enantioselective Diels-Alder reaction catalyzed by Cu^{2+}·ATP with different copper(II) salts.[a]

Entry	Cofactor	Conversion (%)	*Endo*/*exo*	ee (**3a**%, *exo*)	ee (**3a**%, *endo*)
1	$Cu(OTf)_2$	98	91:9	79	72
2	$CuSO_4$	95	90:10	77	67
3	$CuCl_2$	98	91:9	74	62
4	$Cu(NO_3)_2$	90	91:9	74	65

[a] Reaction conditions: **1a** (1 mM), **2** (200 mM), ATP (250 μM), Cu(II) salt (50 μM), MES buffer (20 mM, pH 5.5), 4 ºC, 24 h.

Supplementary Table 2 Enantioselective Diels-Alder reaction catalyzed by Cu^{2+}·ATP with different molar ratios.[a]

Entry	ATP (μM)	$Cu(OTf)_2$ (μM)	Conversion (%)	*Endo*/*exo*	ee (**3a**%, *endo*)
1	500	50	92	91:9	66
2	250	50	98	91:9	72
3	100	50	67	91:9	57
4	50	50	63	91:9	46
5	25	50	83	91:9	20
6	10	50	78	91:9	7
7	5	50	73	92:8	1
8	250	25	72	89:11	66
9	250	50	98	91:9	72
10	250	125	79	91:9	62
11	250	250	67	91:9	52
12	250	500	88	92:8	46

[a] Reaction conditions: **1a** (1 mM), **2** (200 mM), ATP (5-500 μM), $Cu(OTf)_2$ (25-500 μM), MES buffer (20 mM, pH 5.5), 4 ºC, 24 h.

Supplementary Table 3 Enantioselective Diels-Alder reaction catalyzed by Cu^{2+}·ATP with different additives.[a]

Entry	Additive	Concentration (mM)	Conversion (%)	*Endo*/*exo*	ee (**3a**%, *endo*)
1	none	none	98	91:9	72
2	NaCl	10	74	90:10	65
3	KCl	10	87	90:10	67
4	NH_4Cl	10	75	90:10	65
5	$MgCl_2$	10	90	91:9	53
6	$MgCl_2$	50	67	91:9	42
7	$MgCl_2$	100	92	90:10	31
8	$MgCl_2$	250	94	93:7	18
9	$MgCl_2$	500	85	93:7	4
10	$MgCl_2$	1000	87	93:7	0

[a] Reaction conditions: **1a** (1 mM), **2** (200 mM), additive, ATP (250 μM), $Cu(OTf)_2$ (50 μM), MES buffer (20 mM, pH 5.5), 4 ^oC, 24 h.

Supplementary Table 4 Enantioselective Diels-Alder reaction catalyzed by Cu^{2+}·ATP at different temperatures.[a]

Entry	*T* (^oC)	Conversion (%)	*Endo*/*exo*	ee (**3a**%, *endo*)
1	0	87	91:9	66
2	4	98	91:9	72
3	15	98	90:10	62
4	25	96	91:9	56
5	37	71	89:11	44

[a] Reaction conditions: **1a** (1 mM), **2** (200 mM), ATP (250 μM), $Cu(OTf)_2$ (50 μM), MES buffer (20 mM, pH 5.5), 24 h.

Supplementary Table 5 Enantioselective Diels-Alder reaction catalyzed by Cu^{2+}·ATP in different buffers.[a]

Entry	Buffer	pH	Conversion (%)	*Endo*/*exo*	ee (**3a**%, *endo*)
1	MES	5.0	80	90:10	63
2	MES	5.5	98	91:9	72
3	MES	6.0	90	90:10	65
4	MES	6.5	74	90:10	64
5	MOPS	6.5	93	89:11	69
6	MOPS	7.0	89	89:11	67
7	MOPS	7.4	87	88:12	69
8	Tris-HCl	7.4	27	86:14	55
9	PBS	7.4	63	89:11	65

[a] Reaction conditions: **1a** (1 mM), **2** (200 mM), ATP (250 μM), $Cu(OTf)_2$ (50 μM), buffer (20 mM), 4 °C, 24 h.

Supplementary Table 6 Comparable catalytic performances of enantioselective Diels-Alder reactions catalyzed by various metallo-biohybrid catalysts.

Reference	Publication year	Biological scaffold	Metal species	Conversion (%)	*Endo/exo*	ee (*endo*, %)
This work	Not yet	ATP	$Cu(OTf)_2$	85	84:16	84
1	1998	L-abrine	$Cu(NO_3)_2$	>90	>90:10	74
2	2000	Ribozyme	NaCl and $MgCl_2$	Not mentioned	95:5	95
3	2005	Double-stranded DNA	$Cu(NO_3)_2$ and 9-aminoacridine derivative	>80	91:9	53
4	2006	Double-stranded DNA	$Cu(NO_3)_2$ and 4,4'-dimethyl-2,2'-bipyridine	>80	99:1	99
5	2006	Bovine serum albumin	Copper phthalocyanine-3,4',4'',4'''-tetrasulfonic acid tetrasodium salt	91	91:9	98
6	2009	Bovine pancreatic polypeptide	$Cu(NO_3)_2$	73	>95:5	83
7	2010	tHisF	$CuSO_4$	73	93:7	46
8	2010	G-quadruplex DNA	$Cu(NO_3)_2$ and 4,4'-dimethyl-2,2'-bipyridine	>85	95:5	34
9	2012	G-quadruplex DNA	$Cu(NO_3)_2$	99	98:2	74
10	2012	LmrR_M89C-Phen	$Cu(NO_3)_2$	93	95:5	97
11	2013	SCP-2LV83C	$Cu(NO_3)_2$ and phenanthroline derivative	20	88:22	25
12	2013	G-quadruplex DNA	5,10,15,20-Tetrakis(1-methylpyridinium-4-yl)porphyrinatocopper(II) tetraperchlorate	94	97:3	69
13	2014	Cyclic peptides	$Cu(NO_3)_2$	85	96:4	96
14	2015	Cucurbit[8]uril	$Cu(NO_3)_2$	99	97:3	92
15	2015	Lipase	$Cu(NO_3)_2$ and phenanthroline derivative	98	94:6	92
16	2015	G-quadruplex DNA	$Cu(NO_3)_2$ and tertpyridine	>99	98:2	99
17	2016	β-Barrel protein nitrobindin	Cu(II) terpyridyl complexes	22	90:10	Not determined
18	2016	FhuA-terpyridyl	$Cu(NO_3)_2$	69	96:4	0
19	2016	Neocarzinostatin	$Cu(NO_3)_2$ and phenanthroline derivative	98	84:16	0
20	2016	G-tripex DNA	$Cu(NO_3)_2$	99	99:1	64
21	2017	DNA hairpins	$Cu(NO_3)_2$ and 4,4'-dimethyl-2,2'-bipyridine	99	99:1	96
22	2017	β-Barrel protein nitrobindin	$Cu(NO_3)_2$	56	95:5	69
23	2017	αRep A3	$Cu(NO_3)_2$ and phenanthroline derivative	11	86:14	62
24	2017	Double-stranded RNA	$Cu(NO_3)_2$ and 4,4'-dimethyl-2,2'-bipyridine	11	91:9	10
25	2018	HEK-A_{24}	$Cu(NO_3)_2$ and phenanthroline derivative	22	80:20	28
26	2019	mTFP	$Cu(NO_3)_2$	97	93:7	26
27	2019	αRep (A3_A3′)F119C	Terpy−$Cu(NO_3)_2$	15	93:7	52
28	2019	ACCO	$Cu(NO_3)_2$	100	99:1	>99
29	2020	c-di-AMP	$Cu(OTf)_2$	99	97:3	80

Supplementary Figures

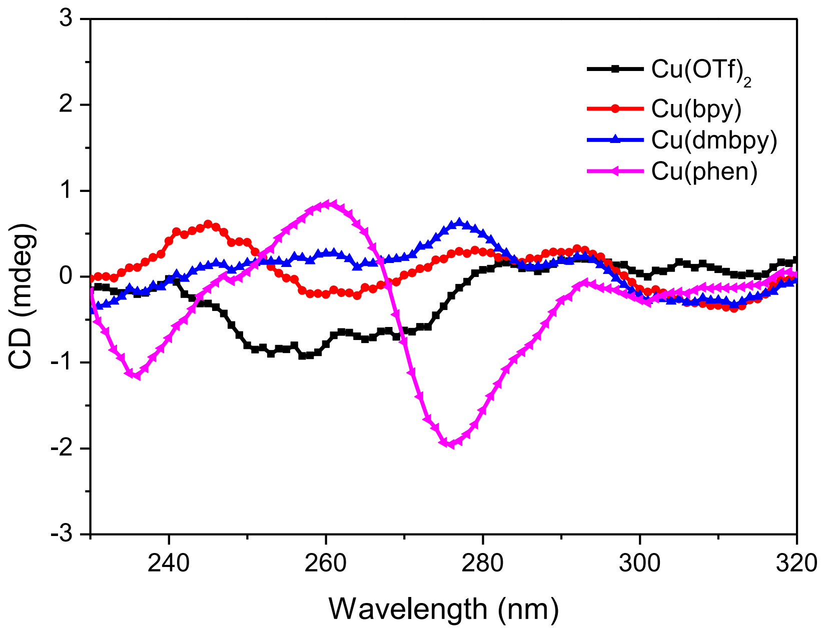

Supplementary Fig. 1 CD spectra of ATP (250 μM) with different copper(II) cofactors (50 μM) in MES buffer (20 mM, pH 5.5).

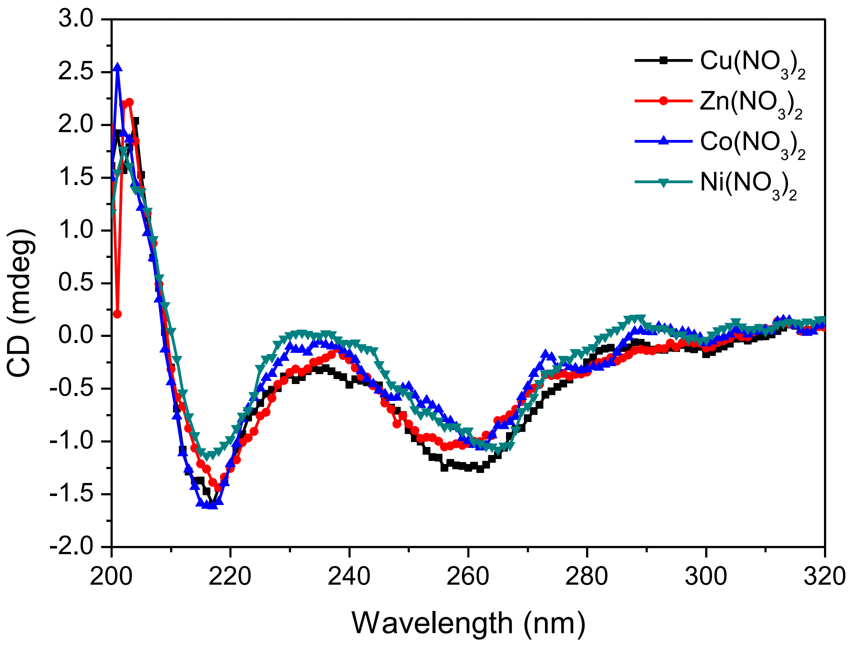

Supplementary Fig. 2 CD spectra of ATP (250 μM) with different divalent metal salts (50 μM) in MES buffer (20 mM, pH 5.5).

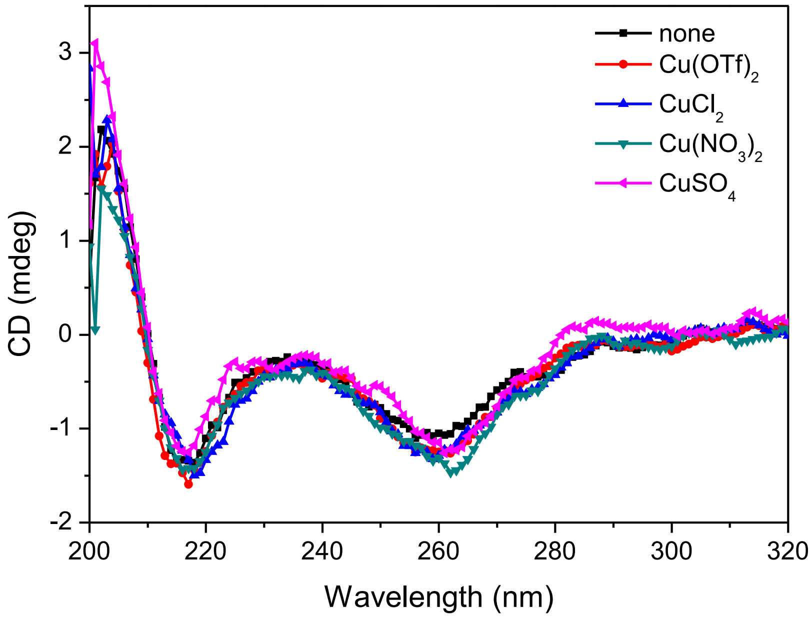

Supplementary Fig. 3 CD spectra of ATP (250 μM) with different copper(II) salts (50 μM) in MES buffer (20 mM, pH 5.5).

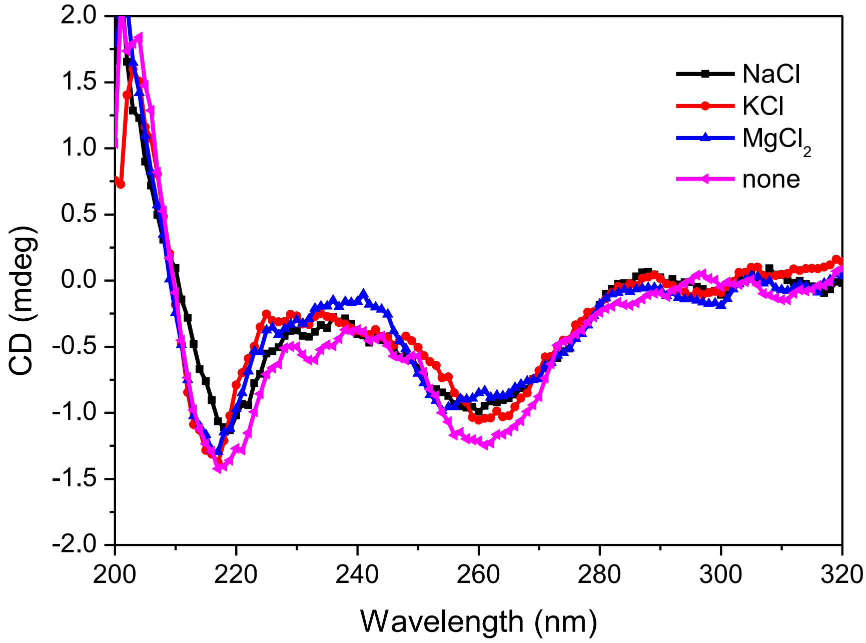

Supplementary Fig. 4 CD spectra of ATP (250 μM) and $Cu(OTf)_2$ (50 μM) in the presence of different additive ions (10 mM) in MES buffer (20 mM, pH 5.5).

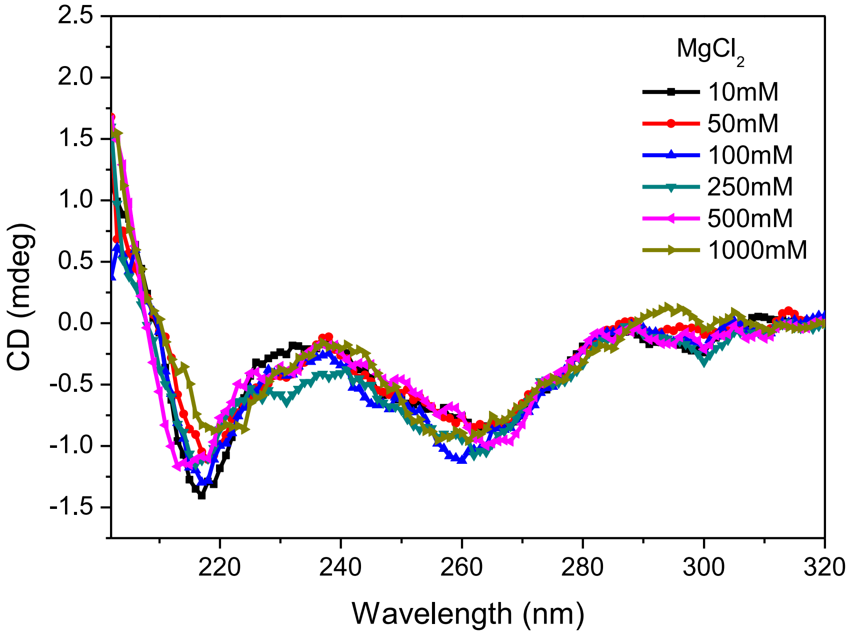

Supplementary Fig. 5 CD spectra of ATP (250 μM) and $Cu(OTf)_2$ (50 μM) with different concentration of $MgCl_2$ (10-1000 mM) in MES buffer (20 mM, pH 5.5).

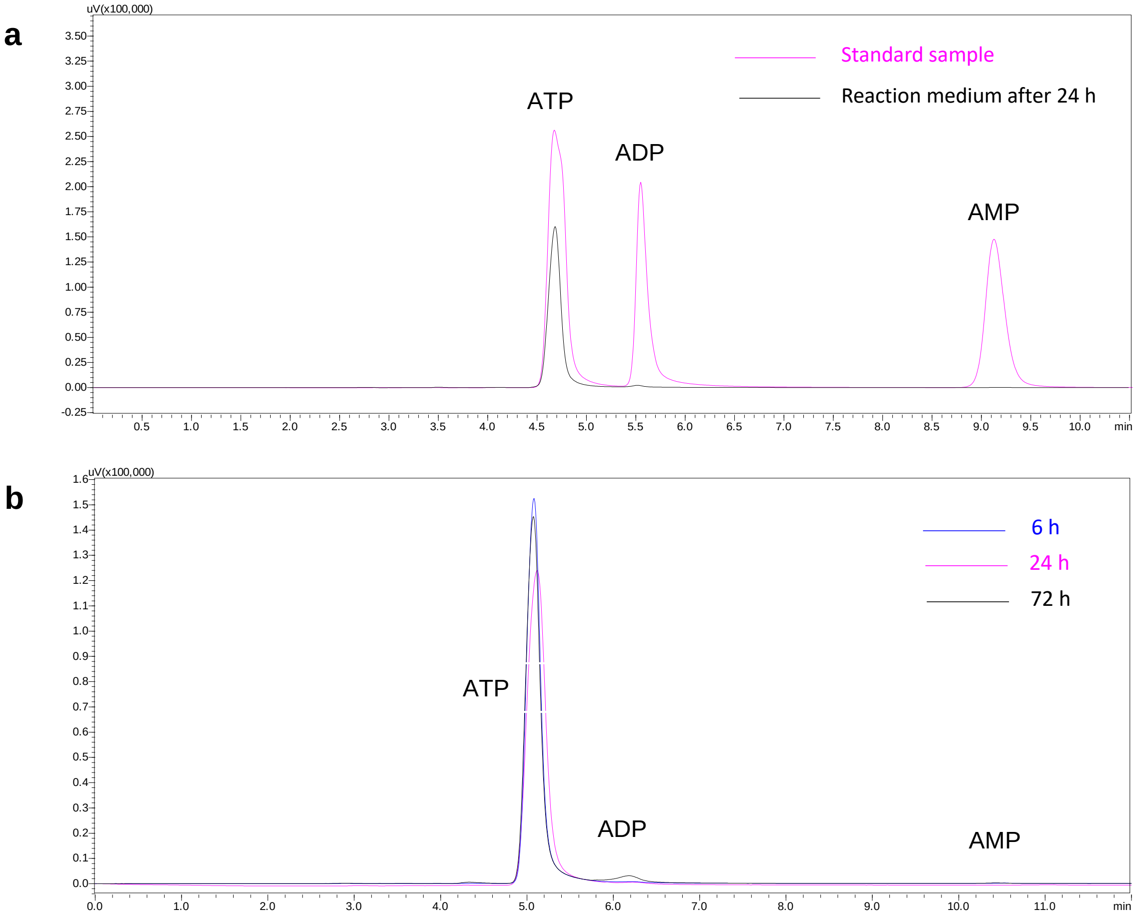

Supplementary Fig. 6 a HPLC analysis of the reaction medium of Cu^{2+}·ATP-catalyzed Diels-Alder reaction. Reaction conditions: **1a** (1 mM), **2** (200 mM), ATP (250 μM), $Cu(OTf)_2$ (50 μM), MES buffer (20 mM, pH 5.5), 4 °C. **b** The stability of ATP in MES buffer (20 mM, pH 5.5) at different reaction time points.

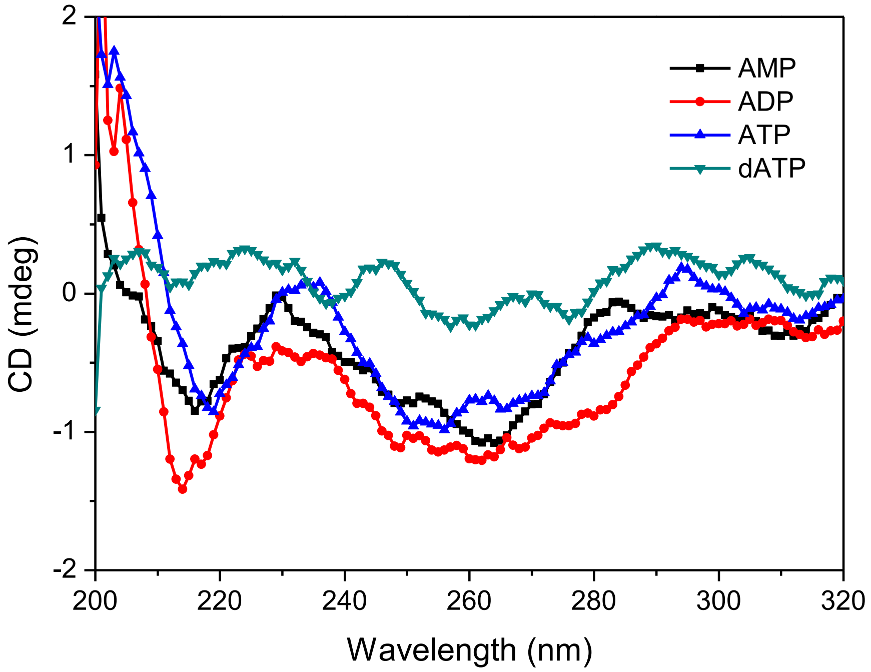

Supplementary Fig. 7 CD spectra of different ATP analogues (250 μM) with $Cu(OTf)_2$ (50 μM) in MES buffer (20 mM, pH 5.5).

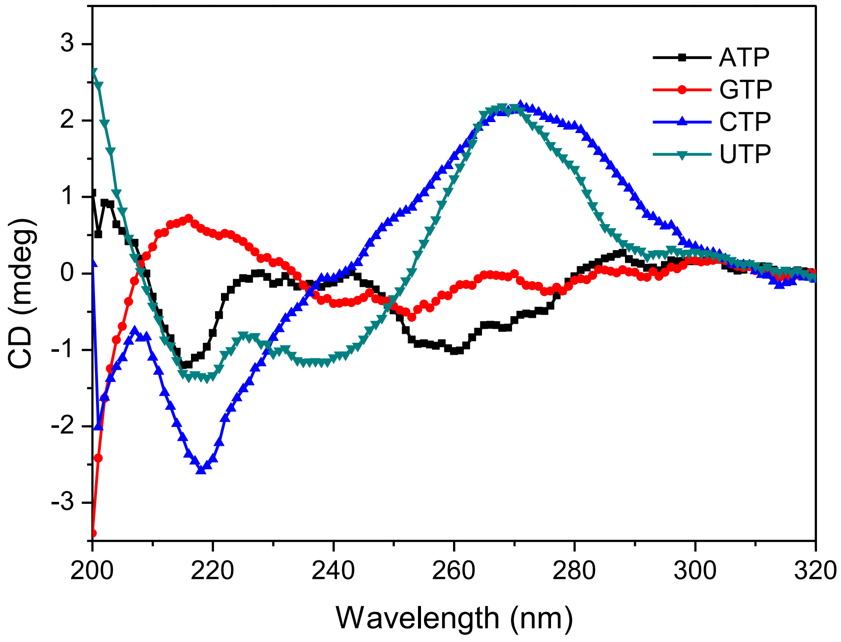

Supplementary Fig. 8 CD spectra of different NTPs (250 μM) with $Cu(OTf)_2$ (50 μM) in MES buffer (20 mM, pH 5.5).

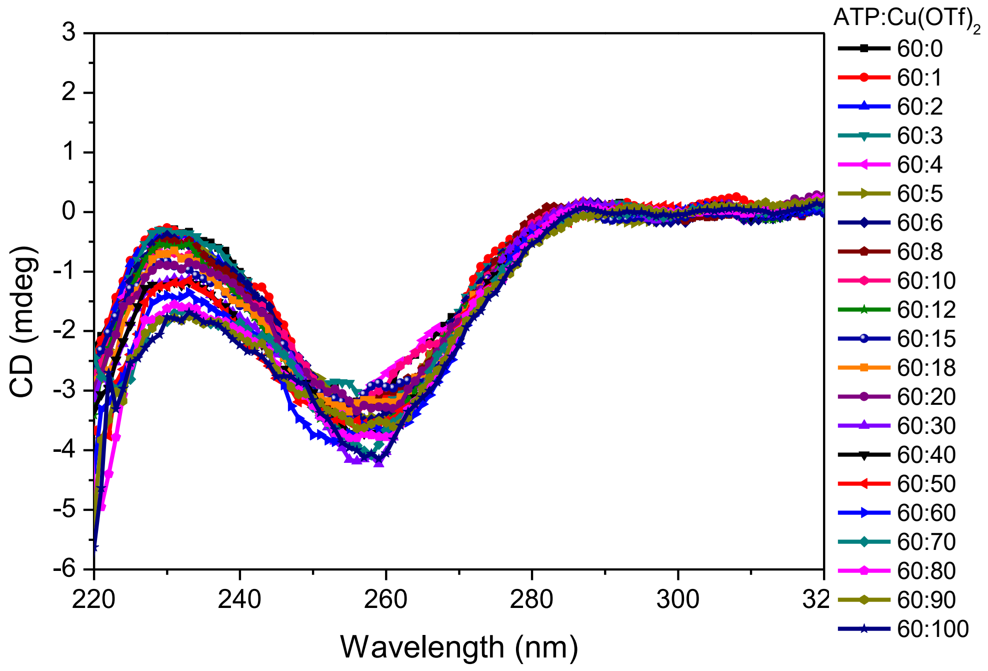

Supplementary Fig. 9 CD spectra of ATP (60 μM) with different amounts of $Cu(OTf)_2$ (0-100 μM) in MES buffer (20 mM, pH 5.5).

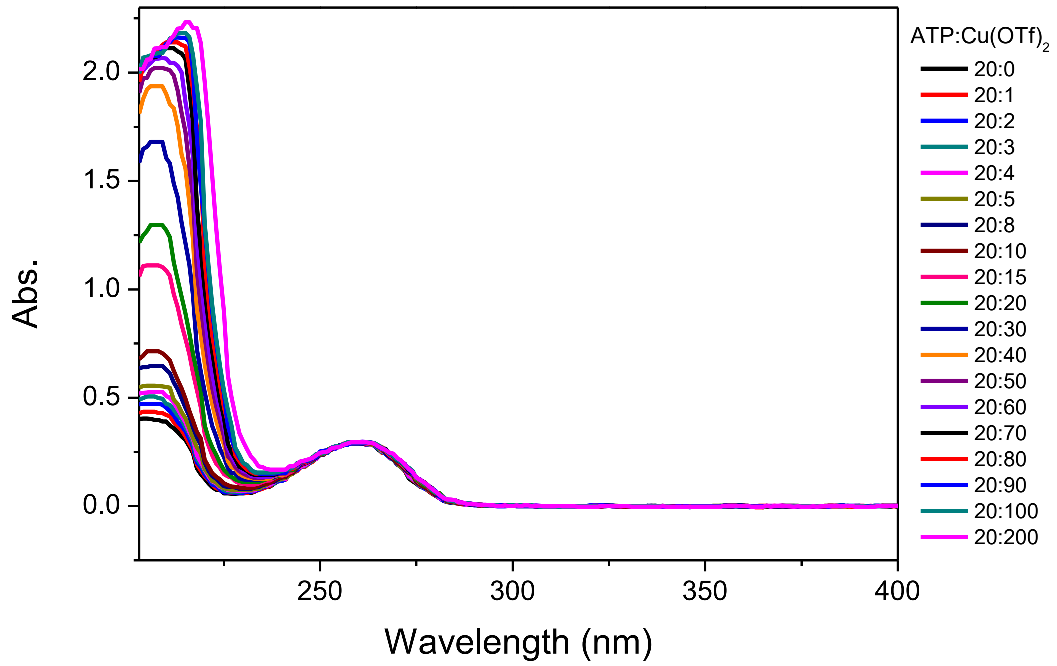

Supplementary Fig. 10 UV-Vis spectra of ATP (20 μM) with different amounts of $Cu(OTf)_2$ (0-200 μM) in MES buffer (20 mM, pH 5.5).

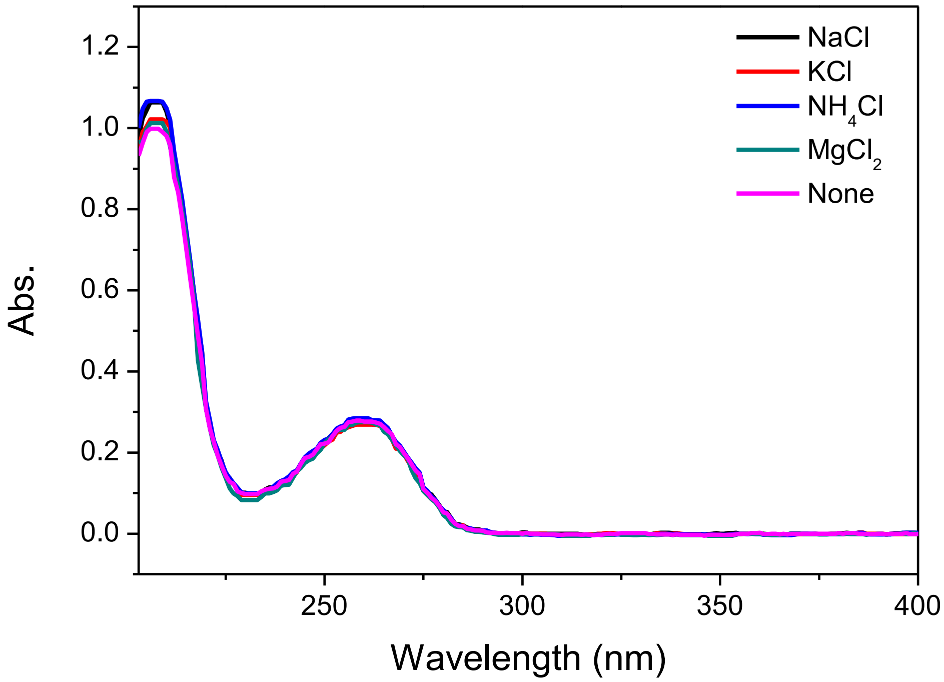

Supplementary Fig. 11 UV-Vis spectra of ATP (20 μM) and $Cu(OTf)_2$ (20 μM) in the presence of different additives (1 mM) in MES buffer (20 mM, pH 5.5).

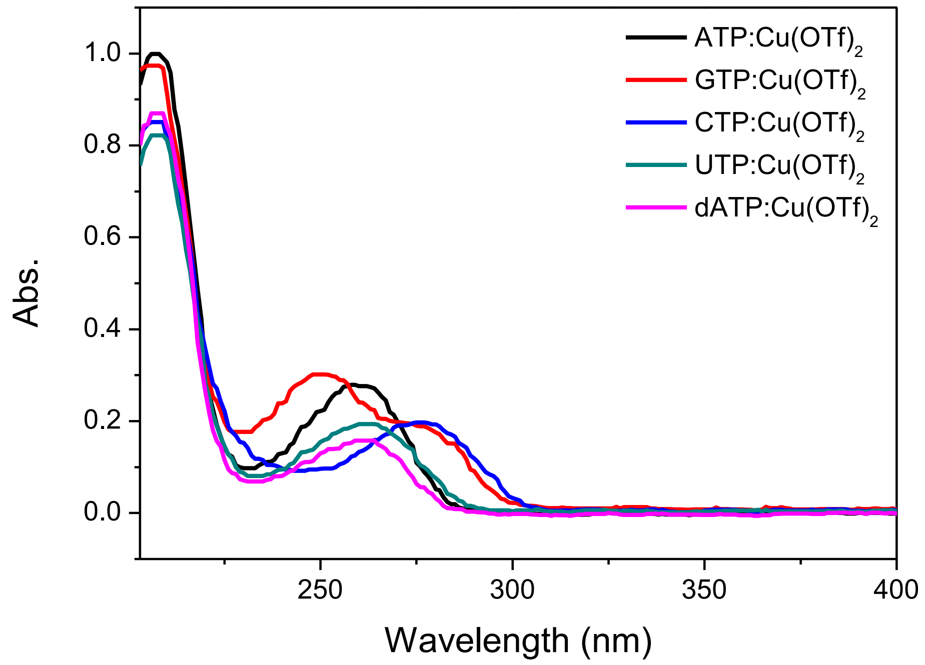

Supplementary Fig. 12 UV-Vis spectra of different NTPs (20 μM) with $Cu(OTf)_2$ (20 μM) in MES buffer (20mM, pH 5.5).

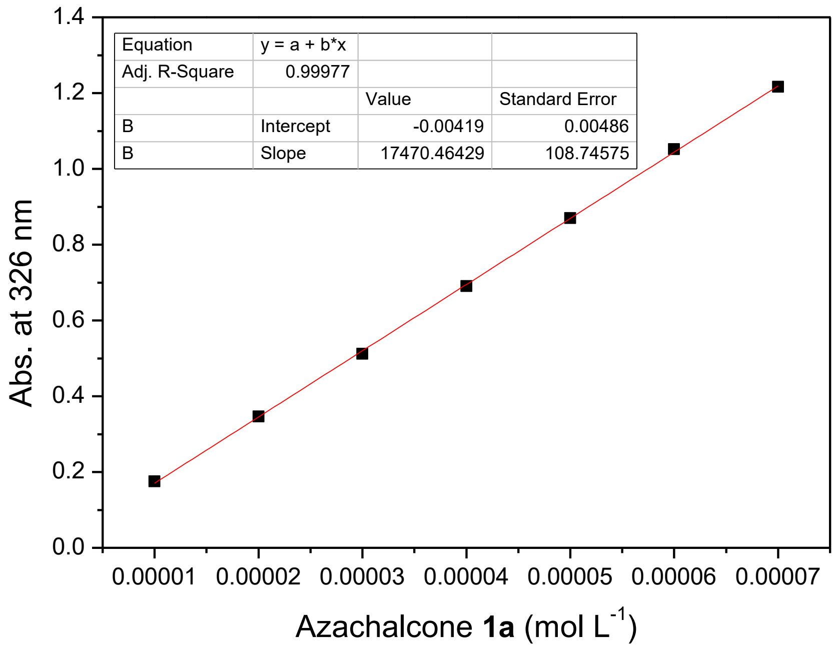

Supplementary Fig. 13 Determination of the molar extinction coefficient of **1a**. The absorbance of **1a** at 326 nm was determined with the fixed concentration of **1a** of 10, 20, 30, 40, 50, 60 and 70 μM. The molar extinction coefficient of **1a** (ε_{1a} = 17470 M^{-1} cm^{-1}) was estimated from the slope of the fitting curve (R^2 = 0.999).

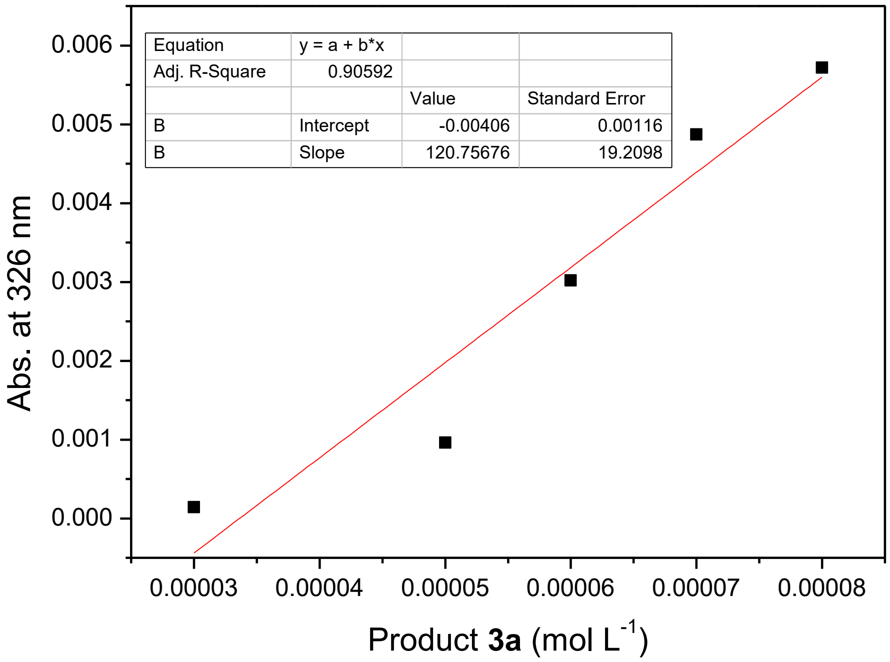

Supplementary Fig. 14 Determination of the molar extinction coefficient of **3a**. The absorbance of **3a** at 326 nm was determined with the fixed concentration of **3a** at 30, 50, 60, 70 and 80 μM. The molar extinction coefficient of **3a** (ε_{3a} = 121 M^{-1} cm^{-1}) was estimated from the slope of the fitting curve (R^2 = 0.906).

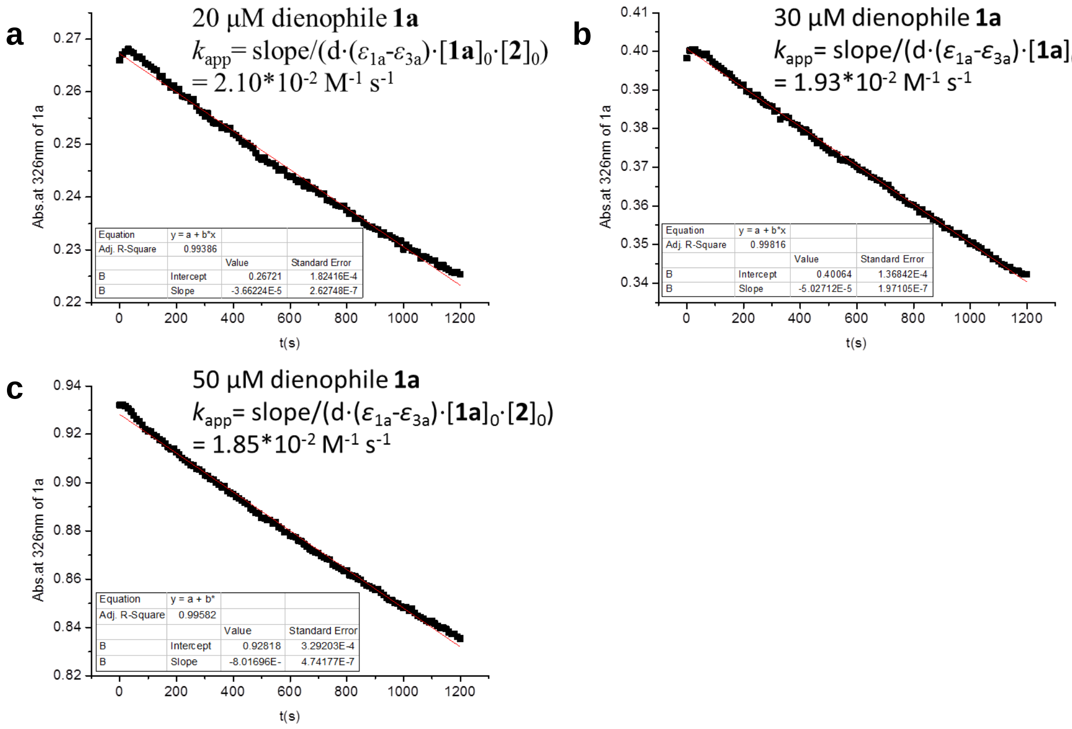

Supplementary Fig. 15 Kinetic plots of Cu^{2+}·ATP catalyzed D-A reactions with dienophile **1a** at the concentrations of **a** 20 μM, **b** 30 μM, and **c** 50 μM. Reaction conditions: **1a** (20, 30, 50 μM), **2** (5 mM), ATP (250 μM), $Cu(OTf)_2$ (50 μM), MES buffer (2000 μL, 20 mM, pH 5.5), 4 °C.

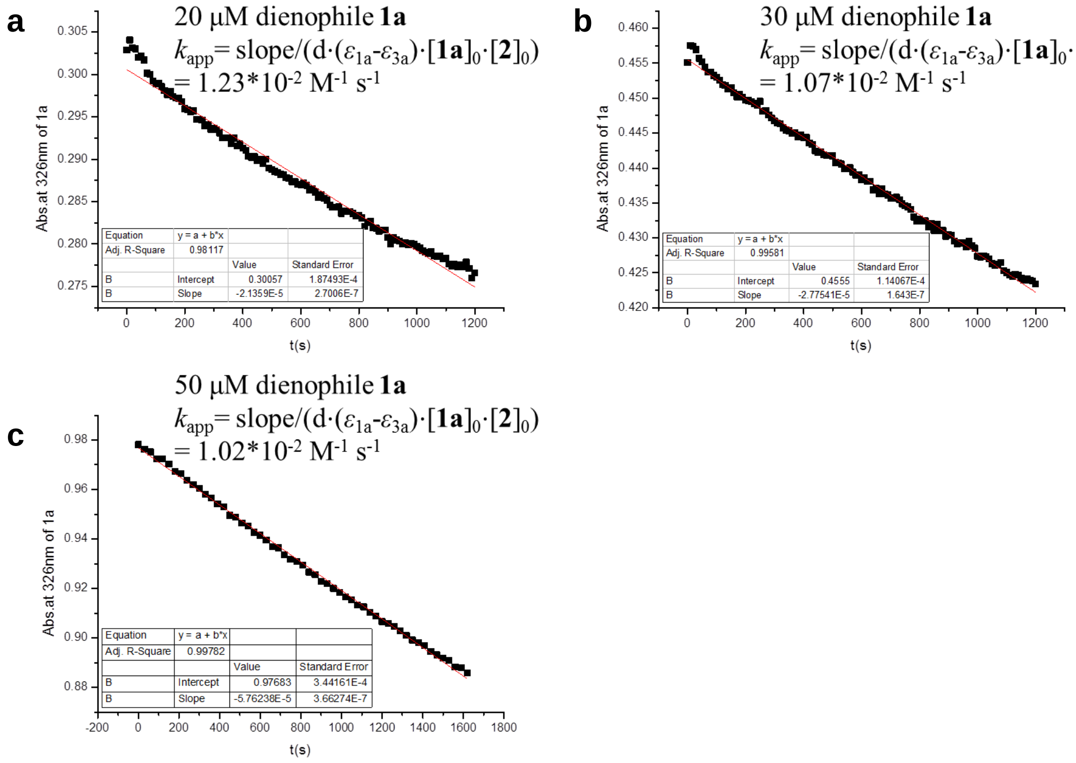

Supplementary Fig. 16 Kinetic plots of $Cu(OTf)_2$ catalyzed D-A reactions with dienophile **1a** at the concentrations of **a** 20 μM, **b** 30 μM, and **c** 50 μM. Reaction conditions: **1a** (20, 30, 50 μM), **2** (5 mM), $Cu(OTf)_2$ (50 μM), MES buffer (2000 μL, 20 mM, pH 5.5), 4 °C.

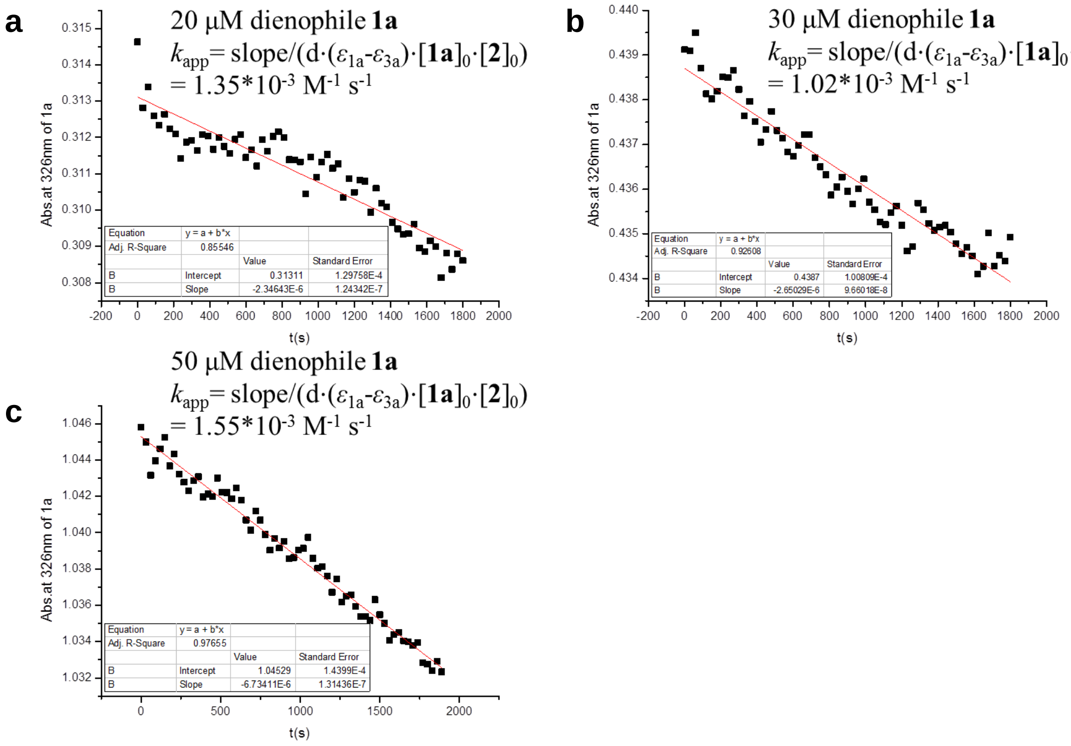

Supplementary Fig. 17 Kinetic plots of ATP catalyzed D-A reactions with dienophile **1a** at the concentrations of **a** 20 μM, **b** 30 μM, and **c** 50 μM. Reaction conditions: **1a** (20, 30, 50 μM), **2** (5 mM), ATP (250 μM), MES buffer (2000 μL, 20 mM, pH 5.5), 4 ºC.

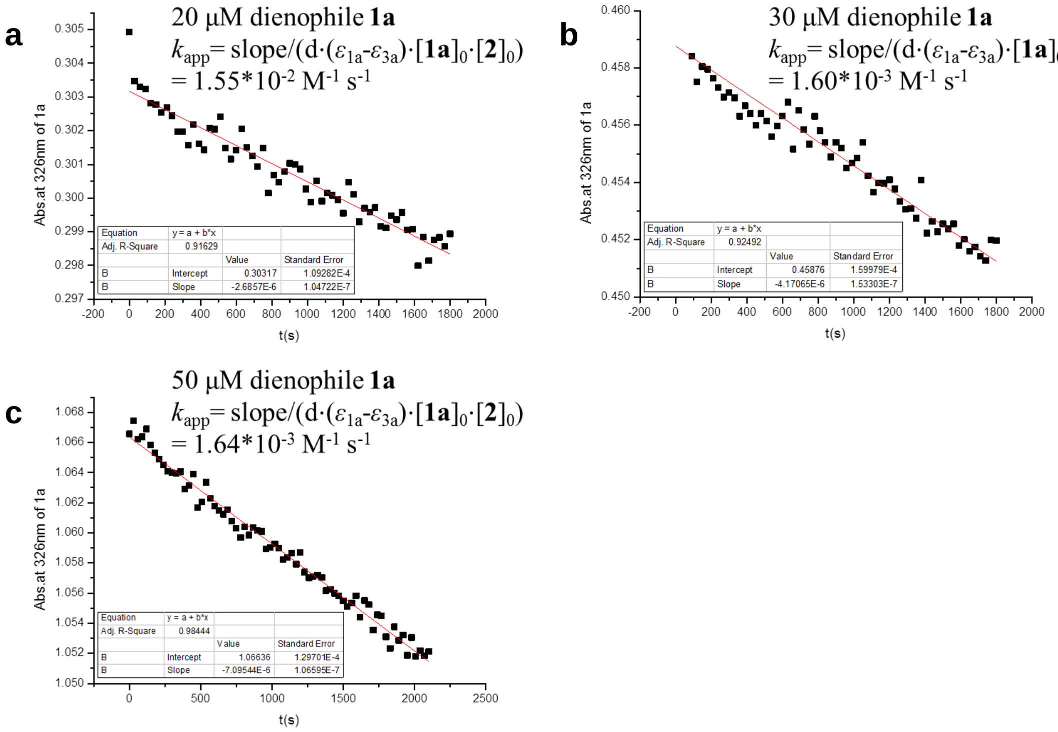

Supplementary Fig. 18 Kinetic plots of the D-A reactions in the absence of catalysts with dienophile **1a** at the concentrations of **a** 20 μM, **b** 30 μM, and **c** 50 μM. Reaction conditions: **1a** (20, 30, 50 μM), **2** (5 mM), MES buffer (2000 μL, 20 mM, pH 5.5), 4 ºC.

Supplementary Methods

General materials. ATP, GTP, UTP, CTP, dATP, ADP, AMP and adenosine were purchased from Sangon (Shanghai, China). The metal salts, achiral ligands and buffers were purchased from Energy Chemical and J&K Scientific Ltd. The chemicals were used without further purification unless otherwise stated. Water used was distilled and deionized using a Milli-Q A10 water purification system. Azachalcones (**1a-h**) and the racemates (**3a-h**) were prepared followed the literature.[30]

Circular dichroism (CD) spectra were measured on a Chirascan circular dichroism spectrometer (Applied Photophysics Ltd, UK). The CD spectra were performed using a quartz cell (1 mm optical path length), an instrument scanning speed of 100 nm min^{-1} and were accumulated by taking the average of three scans made from 200 to 320 nm at 4 °C. UV/Vis spectra were measured on Agilent Cary 3500 or Mettler-Toledo UV5Bio spectrophotometer in a sealed quartz cell with a path length of 1.0 cm. NMR titration spectra (^1H, ^{31}P NMR) of ATP with varied concentration of $CuCl_2$ were performed on a Bruker AV 600 MHz spectrometer. To a solution of ATP (120 mM) in D_2O in a nuclear magnetic tube, a stock solution of $CuCl_2$ in D_2O was gradually added to vary the ratio of ATP/Cu^{2+} from 1000:1 to 5:1. ^1H NMR spectra and ^{31}P NMR spectra were collected at each molar ratio of ATP/Cu^{2+}. Electron paramagnetic resonance (EPR) experiments were conducted on a Bruker EMX plus 10/12 CW X-band EPR spectrometer under a microwave frequency of 9.43 GHz equipped with a liquid nitrogen cooling system. The EPR spectra were measured at 100 K with a modulation amplitude of 0.3 mT, a modulation frequency of 100 kHz, and a microwave power of 20 mW. A mixture of ATP (final conc. 50 mM) and $Cu(OTf)_2$ (final conc. 10 mM) was added to a MES buffer (20 mM, pH 5.5) with glycerol (20 v/v%). After thoroughly mixed, the solution was drawn into a capillary tube and sealed followed by placing into an EPR tube. After cooling in liquid nitrogen for 5 min, the EPR spectra were started to collect.

Determination of the apparent k_b. The apparent binding constant (k_b) of the Cu^{2+}·ATP complex was estimated from the UV titration experiments adopted from the previous reference.[31] To a fixed concentration of $Cu(OTf)_2$ (5 μM) aqueous solution at room temperature, a stock solution of ATP in H_2O was titrated to make the final concentration over the range from 0.1 μM to 100 μM. The apparent k_b for the Cu^{2+}·ATP complex was calculated by the following Supplementary Equation 1:

$$[ATP]/\Delta\varepsilon_a = [ATP]/\Delta\varepsilon + 1/(\Delta\varepsilon_a \cdot k_b) \quad (1)$$

where $\Delta\varepsilon_a = |\varepsilon_{app} - \varepsilon_f|$, $\Delta\varepsilon = |\varepsilon_b - \varepsilon_f|$, ε_{app}, ε_f and ε_b are the apparent, free and bound extinction coefficients for the complex, respectively. In a plot of [ATP]/$\Delta\varepsilon_a$ vs. [ATP], k_b is given by the ratio of the slope to the y intercept.

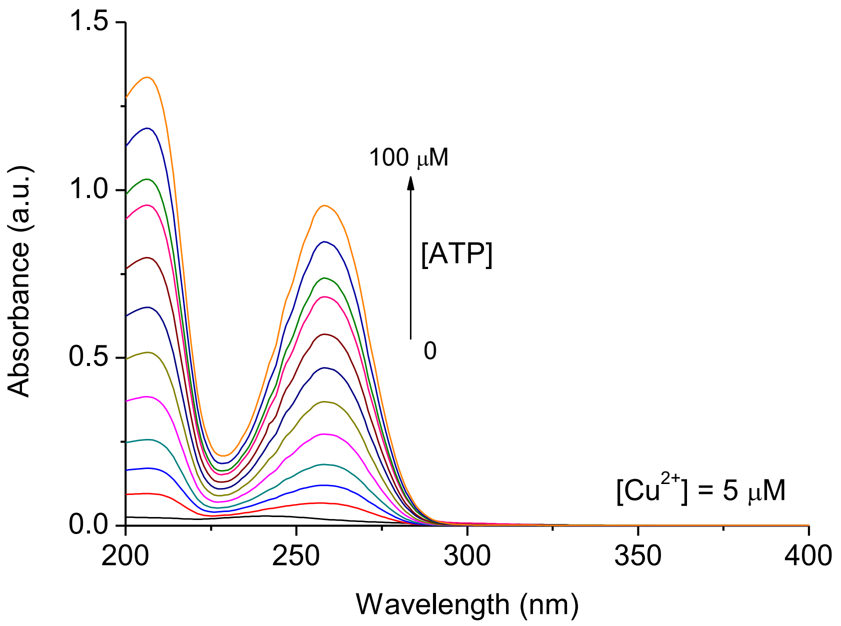

Supplementary Fig. 19 UV titration of varied concentrations of ATP (0-100 μM) to a fixed concentration of $Cu(OTf)_2$ (5 μM) in H_2O.

Determination of the absolute configuration of 3a. For the Cu^{2+}·ATP-catalyzed D-A reaction of **1a** and **2**, the absolute configurations of the product **3a** were determined in comparison with the reported literature.[22] Hayashi et al. reported that the enantioselective D-A reaction of **1a** and **2** was catalyzed by NB-Pyr and Cu(II) ions, yielding the chiral product **3a** (*Si-endo*) at 78% ee in *(1R,2S,3S,4S)* configuration. The *endo* and *exo* isomers of **3a** were assigned on the HPLC trace using Chiralpak IA column (hexane/*i*-PrOH = 99.5:0.5, 0.5 mL min^{-1}, 264 nm) with the order of the **3a** enantiomers of **3a** (*Re-exo*), **3a** (*Si-exo*), **3a** (*Si-endo*) and **3a** (*Re-endo*).[22] Using the same HPLC condition and Chiralpak IA column, we analyzed the product **3a** that was obtained from the Diels–Alder reaction of **1a** and **2** catalyzed by Cu^{2+}·ATP. The HPLC traces of **3a** in racemic form and chiral form are shown in Supplementary Fig. 21. By comparison to the reference, we determined that the major product **3a** generated by Cu^{2+}·ATP was **3a** (*Si-endo*) in *(1R,2S,3S,4S)* configuration.

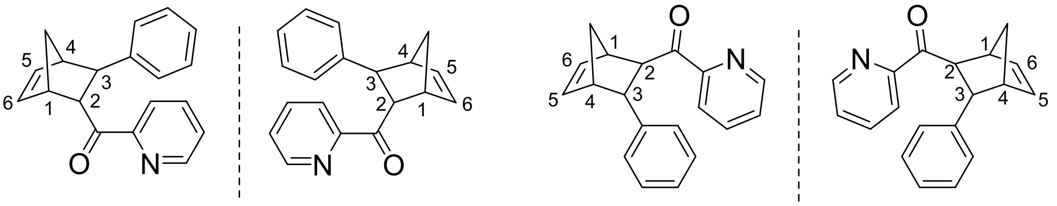

3a (*Re-endo*) 1*S*, 2*R*, 3*R*, 4*R* | **3a** (*Si-endo*) 1*R*, 2*S*, 3*S*, 4*S* | **3a** (*Re-exo*) 1*R*, 2*R*, 3*R*, 4*S* | **3a** (*Si-exo*) 1*S*, 2*S*, 3*S*, 4*R*

Supplementary Fig. 20 The absolute configurations of the D-A product **3a** for the *endo* and *exo* isomers.

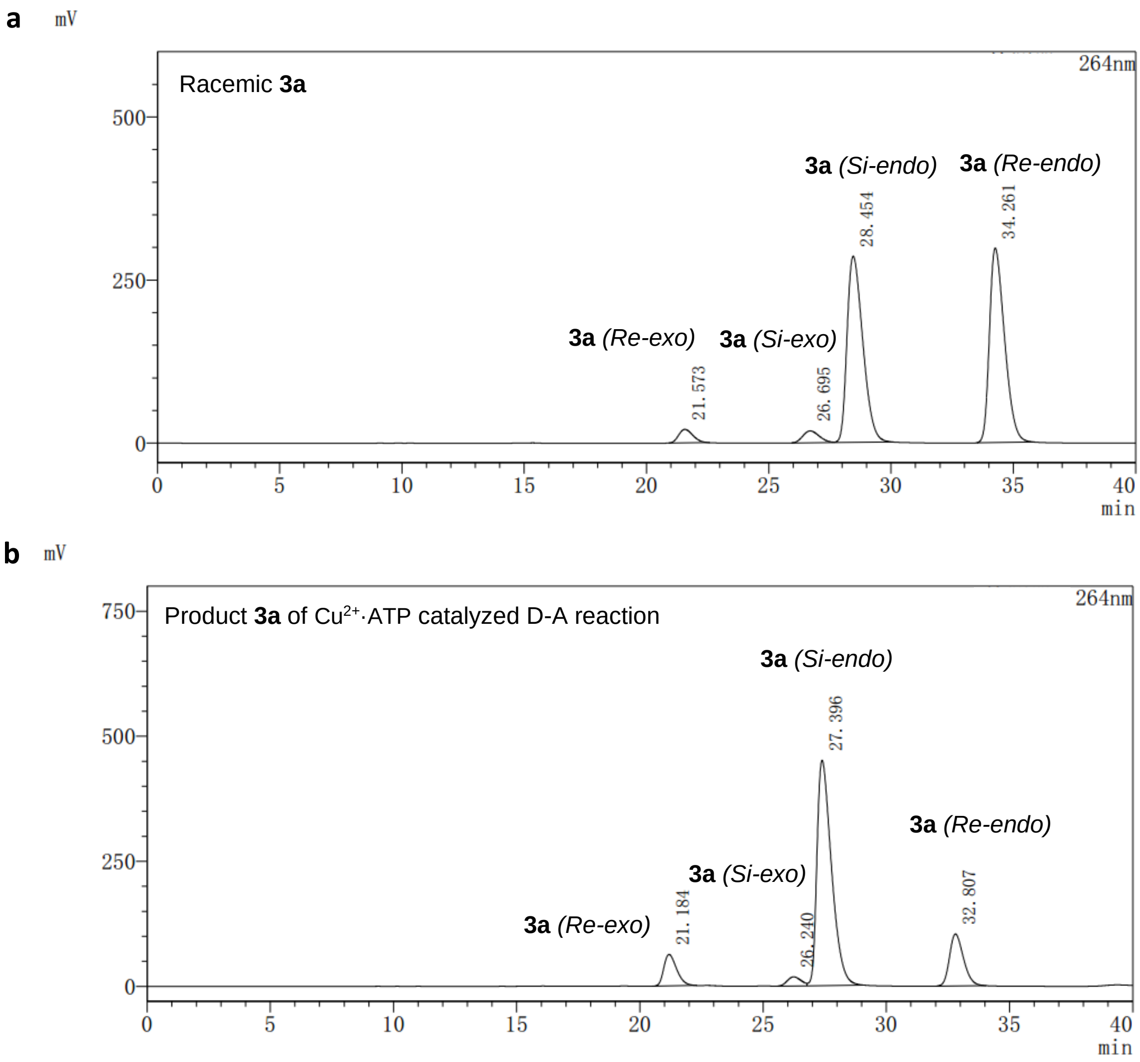

Supplementary Fig. 21 HPLC traces of **a** racemic and **b** product of **3a** from the Diels-Alder reaction catalyzed by Cu^{2+}·ATP using Chiralpak IA column. The major product **3a** generated by Cu^{2+}·ATP is **3a** (*Si-endo*) in *(1R,2S,3S,4S)* configuration.

DFT calculations. In order to compare the stability among all candidates, the relative electronic energies were listed in Supplementary Figs. 22-25 in which the most stable configuration was set to zero.

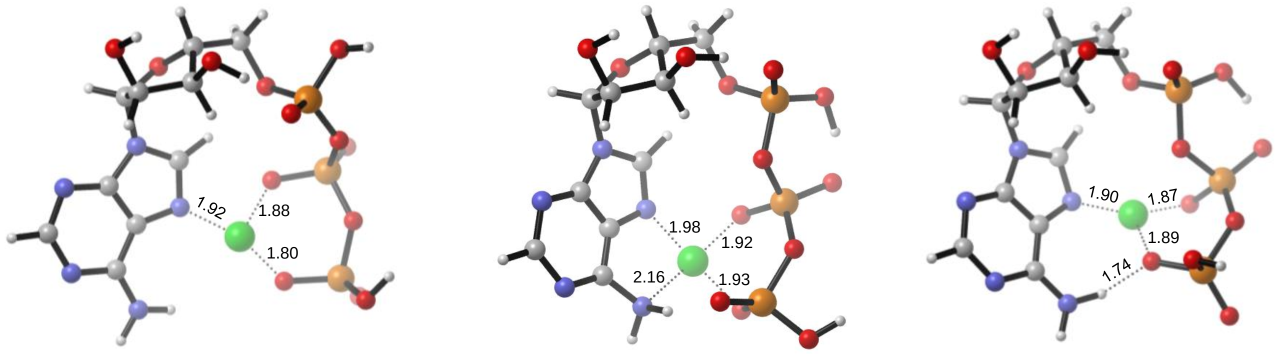

Model 1	Model 2	Model 3
(8.7)	(0.3)	(0.0)

Supplementary Fig. 22 The proposed models of Cu^{2+}·ATP. The relative electronic energies are in the levels of B3LYP-D3/6-311G(d,p)~LANL2DZ with a unit of kcal mol^{-1}.

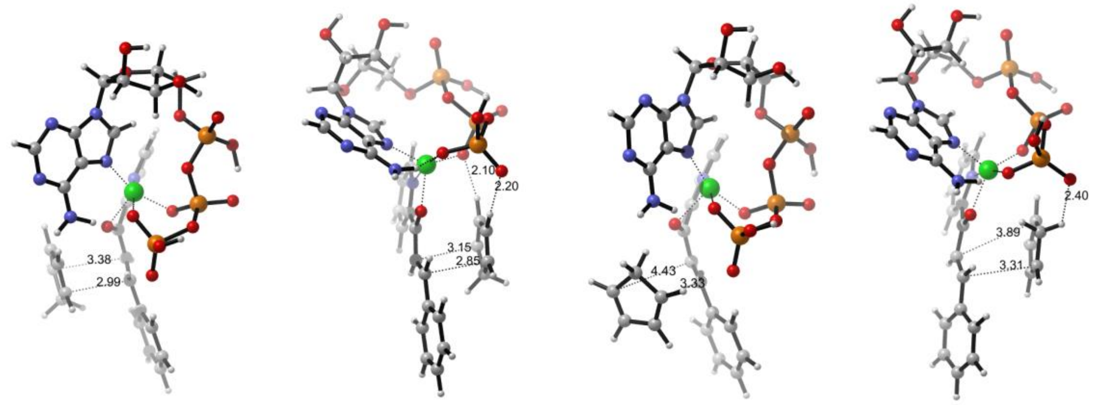

Precusor	**3a** (*Re-endo*)	**3a** (*Si-endo*)	**3a** (*Re-exo*)	**3a** (*Si-exo*)
Δ*E* (kcal mol^{-1})	9.1	0.0	6.4	0.8

Supplementary Fig. 23 The precursors of the intermediates of **1a**-Cu^{2+}·ATP and **2** that yield the corresponding products **3a** in different configurations. The relative electronic energies (Δ*E*) of the precursors are shown in the table.

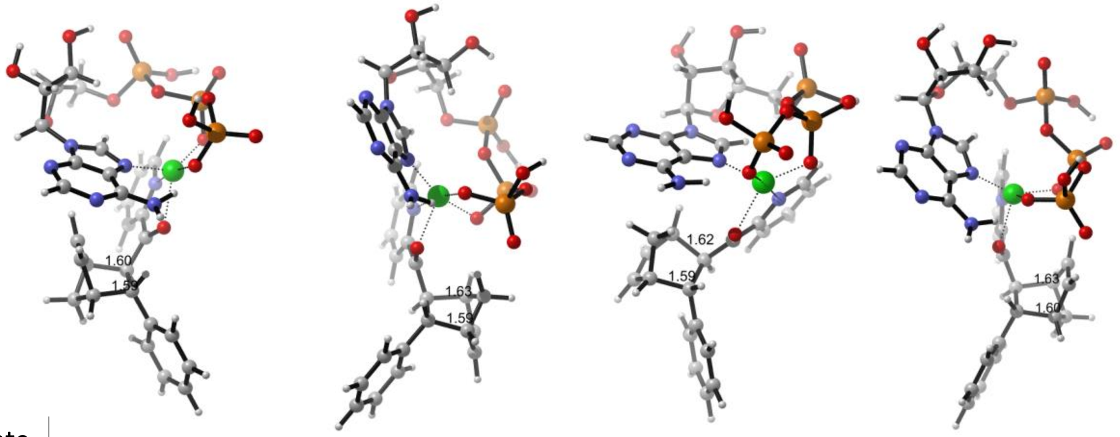

The intermidiate of **3a**-Cu^{2+}·ATP	**3a** (*Re-endo*)	**3a** (*Si-endo*)	**3a** (*Re-exo*)	**3a** (*Si-exo*)
Δ*E* (kcal mol^{-1})	0.9	0.0	-0.5	2.6

Supplementary Fig. 24 The intermediates of **3a**-Cu^{2+}·ATP and their relative electronic energies (Δ*E*).

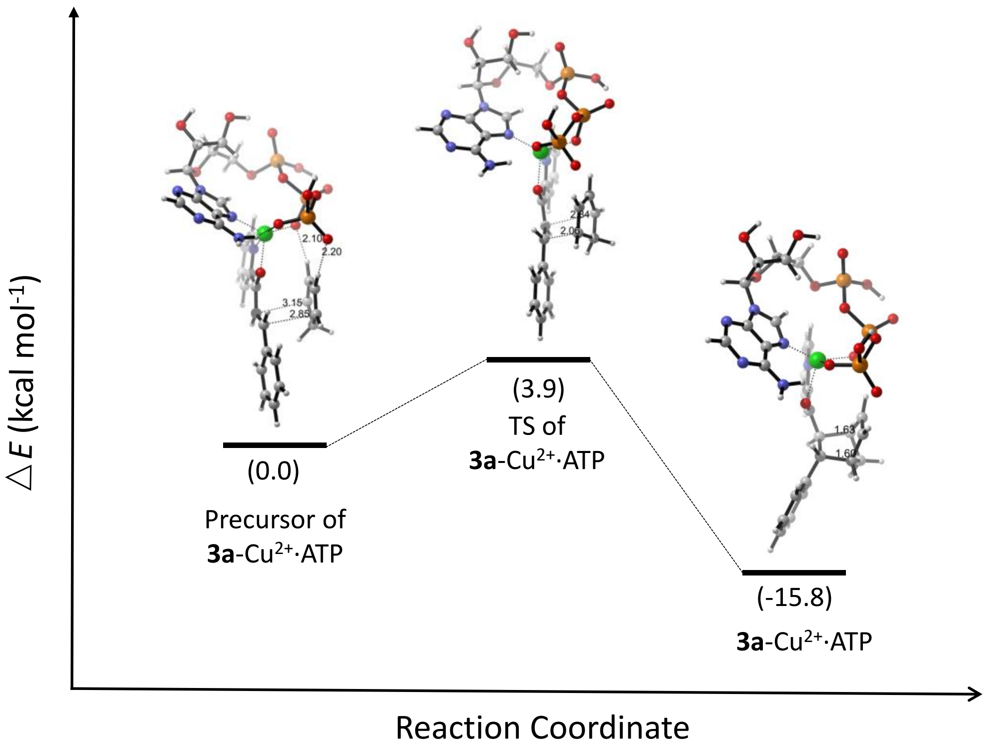

Supplementary Fig. 25 The relative electronic energy profile of the reaction path for Cu^{2+}·ATP-catalyzed Diels-Alder reaction of **1a** and **2** that yields **3a** (*endo*) in the absolute configuration of 1*R*, 2*S*, 3*S*, 4*S*. Abbreviation: TS, transition state.

HPLC analysis. The enantioselectivity of **3a-h** was determined by a chiral HPLC column (250 × 4.6 mm) with hexane and *i*-PrOH as eluents.

a

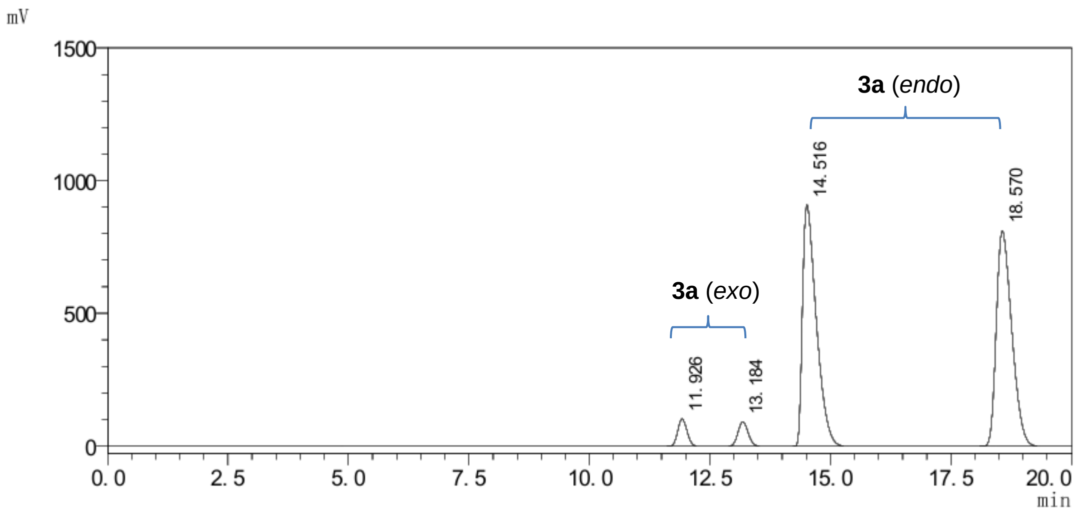

<Peak table>

Peak#	Ret. Time	Area	Height	Conc.	Unit	Mark	Name
1	11. 926	1350700	102932	0. 000		M	
2	13. 184	1342646	91959	0. 000		M	
3	14. 516	18082841	909607	0. 000		M	
4	18. 570	18106792	811480	0. 000		M	

b

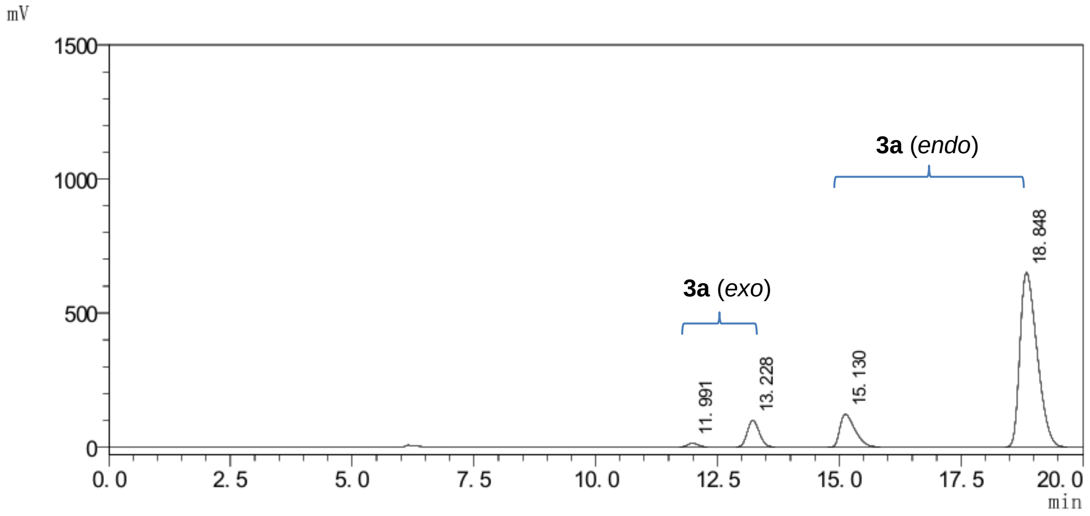

<Peak table>

Peak#	Ret. Time	Area	Height	Conc.	Unit	Mark	Name
1	11. 991	197582	13485	0. 000		M	
2	13. 228	1691906	99167	0. 000		M	
3	15. 130	2615605	122022	0. 000		M	
4	18. 848	16044452	651347	0. 000		M	

Supplementary Fig. 26 a HPLC trace of racemic **3a**. **b** HPLC trace of prodcut **3a** that from the Diels-Alder reaction catalyzed by Cu^{2+}·ATP for the *exo* isomer of 79% ee and the *endo* isomer of 72% ee. HPLC condition: Daicel Chiralpak ODH column, hexane/*i*-PrOH 98:2, 0.5 mL min^{-1}, 254 nm, 25 °C.

a

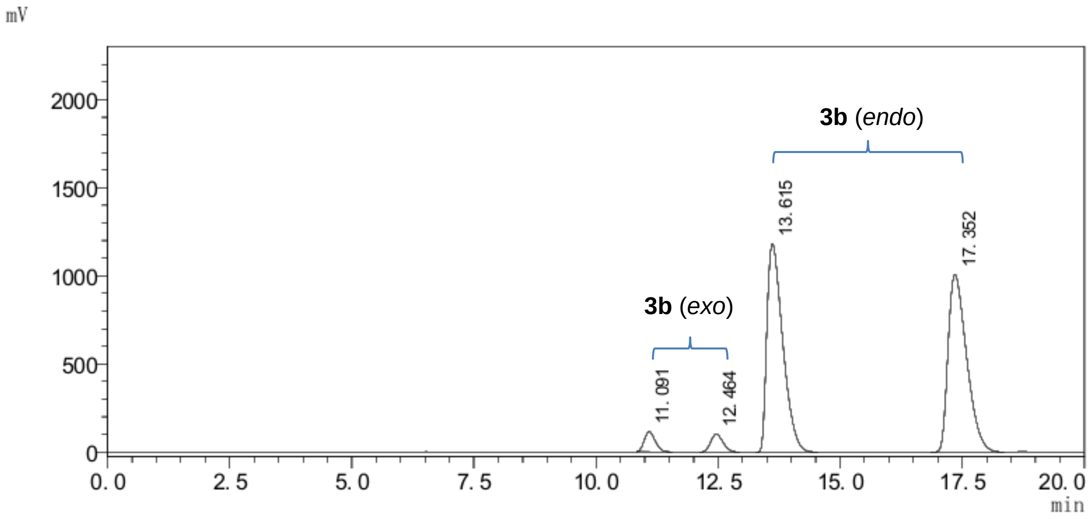

<Peak table>

Peak#	Ret. Time	Area	Height	Conc.	Unit	Mark	Name
1	11. 091	1780210	113474	0. 000		M	
2	12. 464	1809308	101959	0. 000		M	
3	13. 615	26721126	1184550	0. 000		M	
4	17. 352	26727757	1008109	0. 000		M	

b

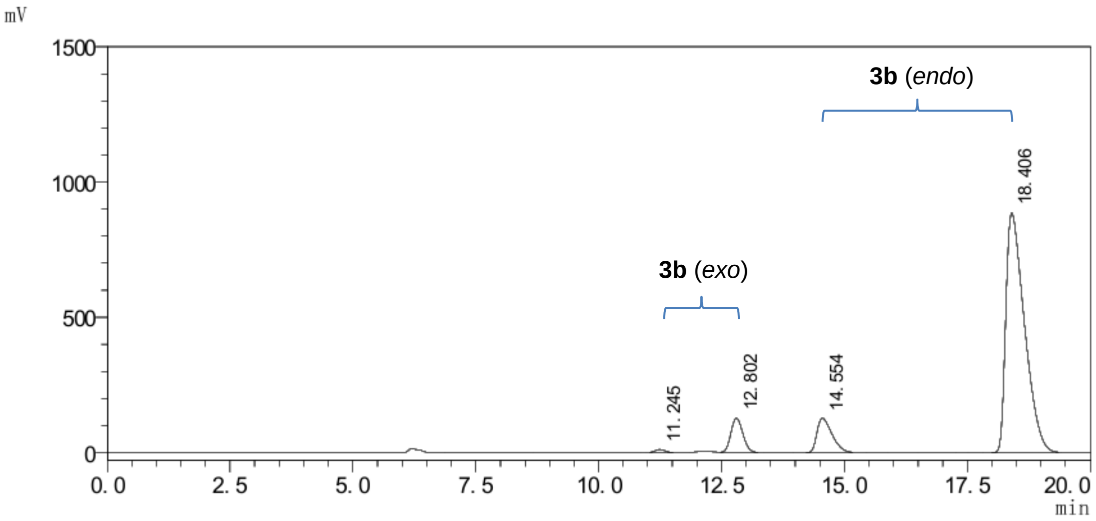

<Peak table>

Peak#	Ret. Time	Area	Height	Conc.	Unit	Mark	Name
1	11. 245	136723	10191	0. 000		M	
2	12. 802	2111972	126052	0. 000		M	
3	14. 554	2652252	126124	0. 000		M	
4	18. 406	23358557	885340	0. 000		M	

Supplementary Fig. 27 a HPLC trace of racemic **3b**. **b** HPLC trace of product **3b** from the Diels-Alder reaction catalyzed by Cu^{2+}·ATP for the *exo* isomer of 88% ee and the *endo* isomer of 80% ee. HPLC condition: Daicel Chiralpak OD column, hexane/*i*-PrOH 98:2, 0.5 mL min^{-1}, 254 nm, 25 °C.

a

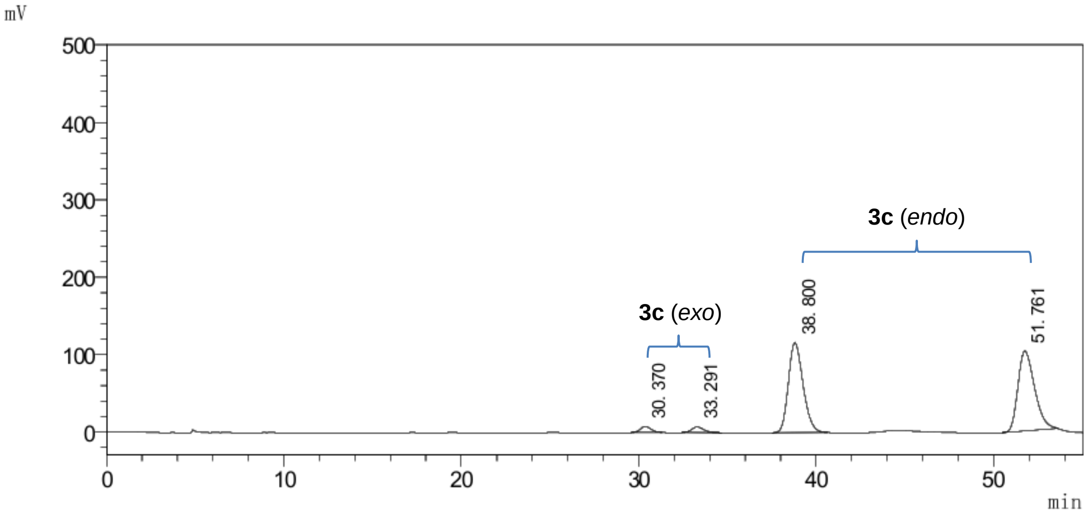

<Peak table>

Peak#	Ret. Time	Area	Height	Conc.	Unit	Mark	Name
1	30. 370	326071	7867	0. 000		M	
2	33. 291	334338	7125	0. 000		M	
3	38. 800	6685877	116095	0. 000		M	
4	51. 761	6736538	103189	0. 000		M	

b

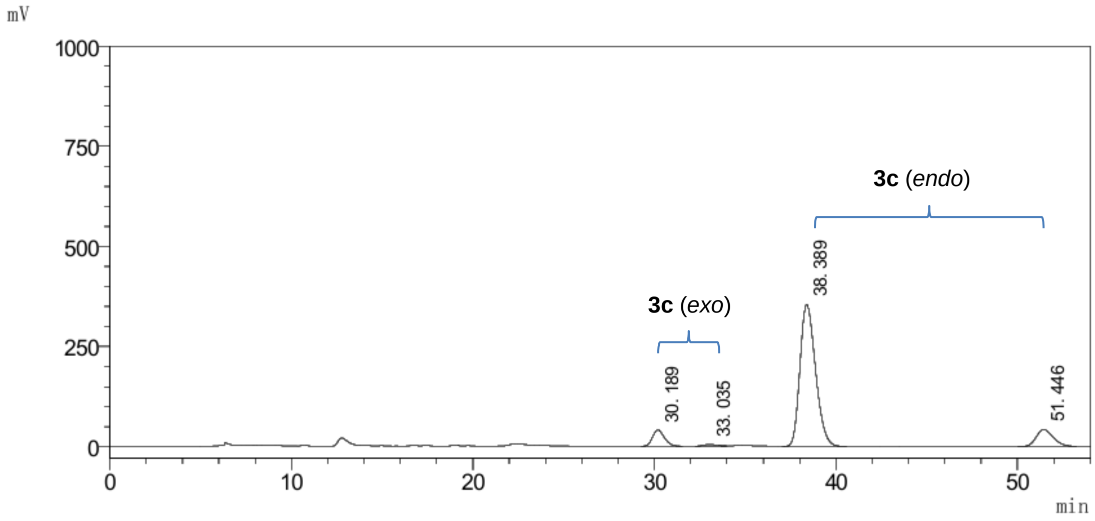

<Peak table>

Peak#	Ret. Time	Area	Height	Conc.	Unit	Mark	Name
1	30. 189	1834231	40402	0. 000		M	
2	33. 035	199267	4213	0. 000		M	
3	38. 389	21151294	354664	0. 000		M	
4	51. 446	2745824	41898	0. 000		M	

Supplementary Fig. 28 a HPLC trace of racemic **3c**. **b** HPLC trace of product **3c** from the Diels-Alder reaction catalyzed by Cu^{2+}·ATP for the *exo* isomer of 80% ee and the *endo* isomer of 77% ee. HPLC condition: Daicel Chiralpak AD column, hexane/*i*-PrOH 98:2, 0.5 mL min^{-1}, 254 nm, 25 ^{o}C.

a

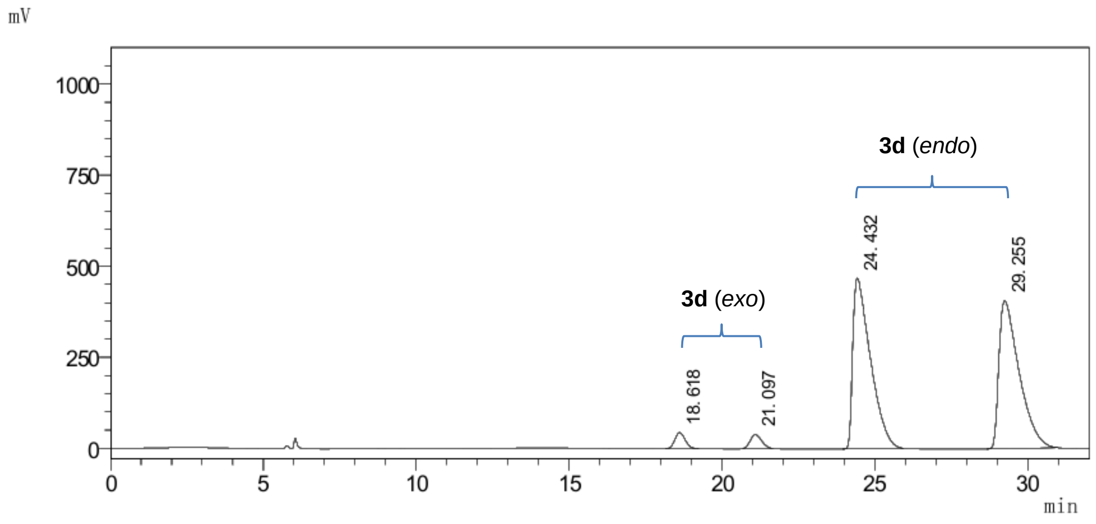

<Peak table>

Peak#	Ret. Time	Area	Height	Conc.	Unit	Mark	Name
1	18. 618	1041998	44316	0. 000		M	
2	21. 097	983607	37988	0. 000		M	
3	24. 432	18842320	468209	0. 000		M	
4	29. 255	18676896	406218	0. 000		M	

b

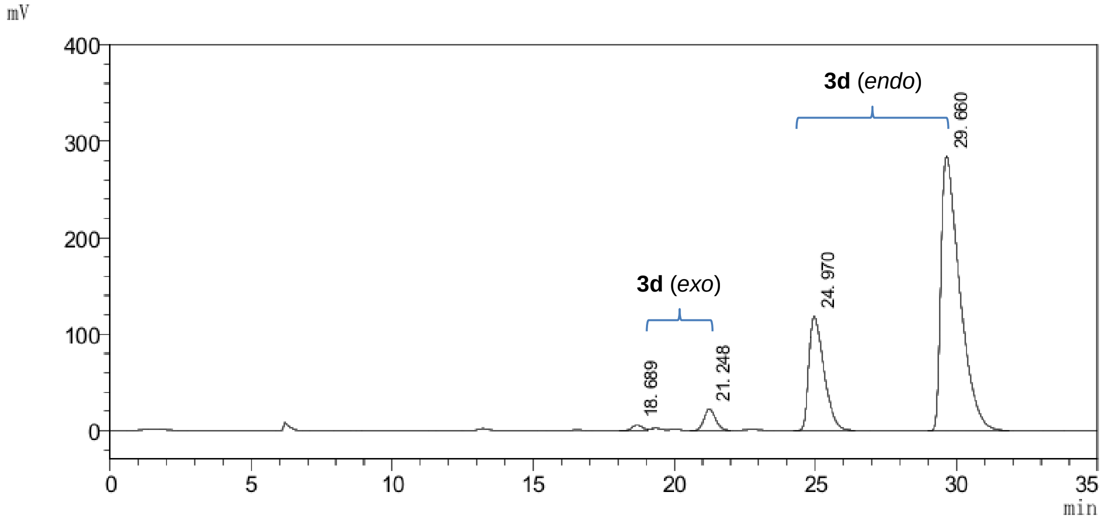

<Peak table>

Peak#	Ret. Time	Area	Height	Conc.	Unit	Mark	Name
1	18. 689	144639	5613	0. 000		M	
2	21. 248	625606	21924	0. 000		M	
3	24. 970	4390140	118597	0. 000		M	
4	29. 660	13248237	284312	0. 000		M	

Supplementary Fig. 29 a HPLC trace of racemic **3d**. **b** HPLC trace of product **3d** from the Diels-Alder reaction catalyzed by Cu^{2+}·ATP for the *exo* isomer of 62% ee and the *endo* isomer of 50% ee. HPLC condition: Daicel Chiralpak ODH column, hexane/*i*-PrOH 98:2, 0.5 mL min^{-1}, 254 nm, 25 °C.

a

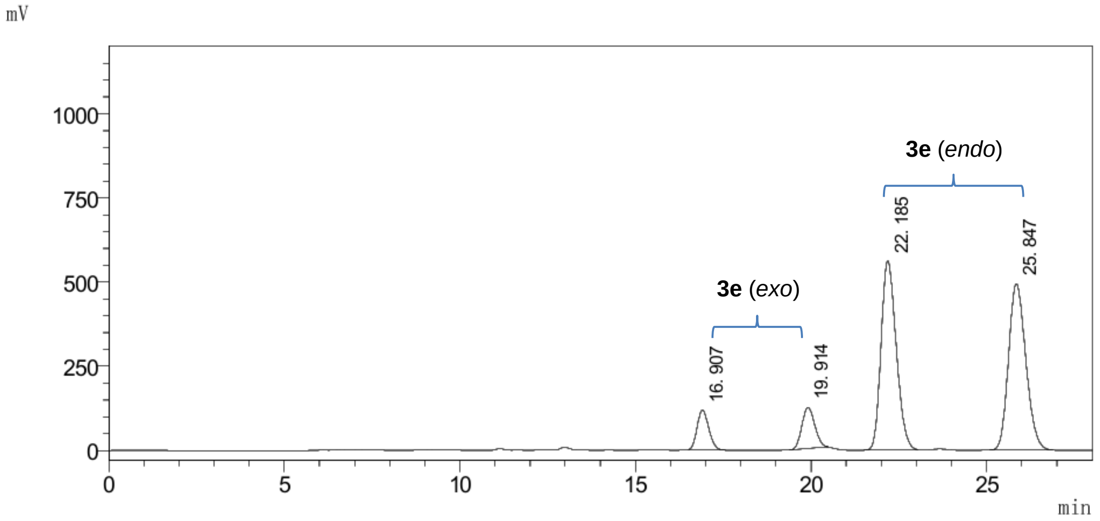

<Peak table>

Peak#	Ret. Time	Area	Height	Conc.	Unit	Mark	Name
1	16. 907	2717591	117947	0. 000		M	
2	19. 914	3118196	121946	0. 000		M	
3	22. 185	16864839	561311	0. 000		M	
4	25. 847	16970924	494182	0. 000		M	

b

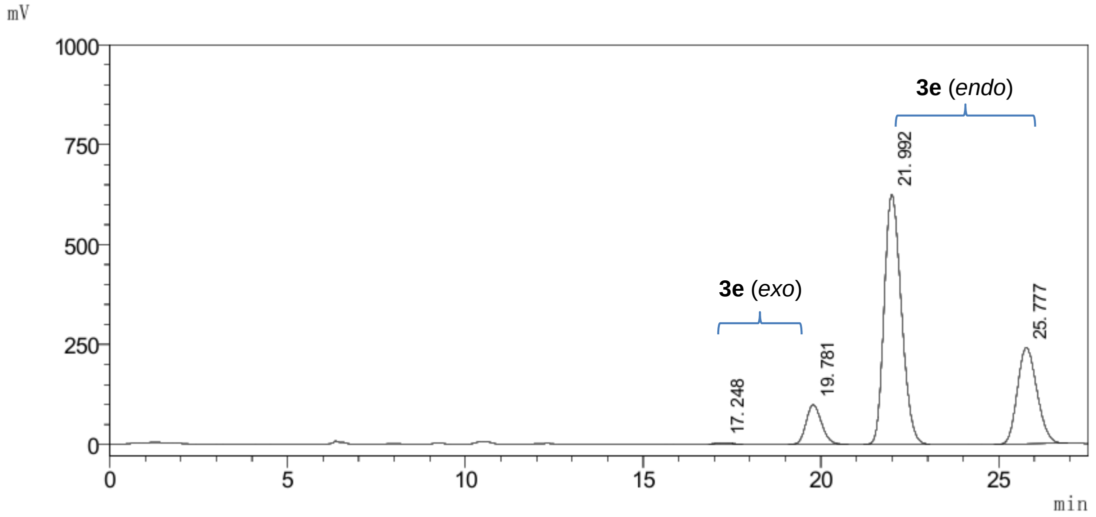

<Peak table>

Peak#	Ret. Time	Area	Height	Conc.	Unit	Mark	Name
1	17. 248	108089	4005	0. 000		M	
2	19. 781	2974872	98890	0. 000		M	
3	21. 992	20989034	625065	0. 000		M	
4	25. 777	9242805	241024	0. 000		M	

Supplementary Fig. 30 a HPLC trace of racemic **3e**. **b** HPLC trace of product **3e** from the Diels-Alder reaction catalyzed by Cu^{2+}·ATP for the *exo* isomer of 93% ee and the *endo* isomer of 39% ee. HPLC condition: Daicel Chiralpak AD column, hexane/*i*-PrOH 98:2, 0.5 mL min^{-1}, 254 nm, 25 °C.

a

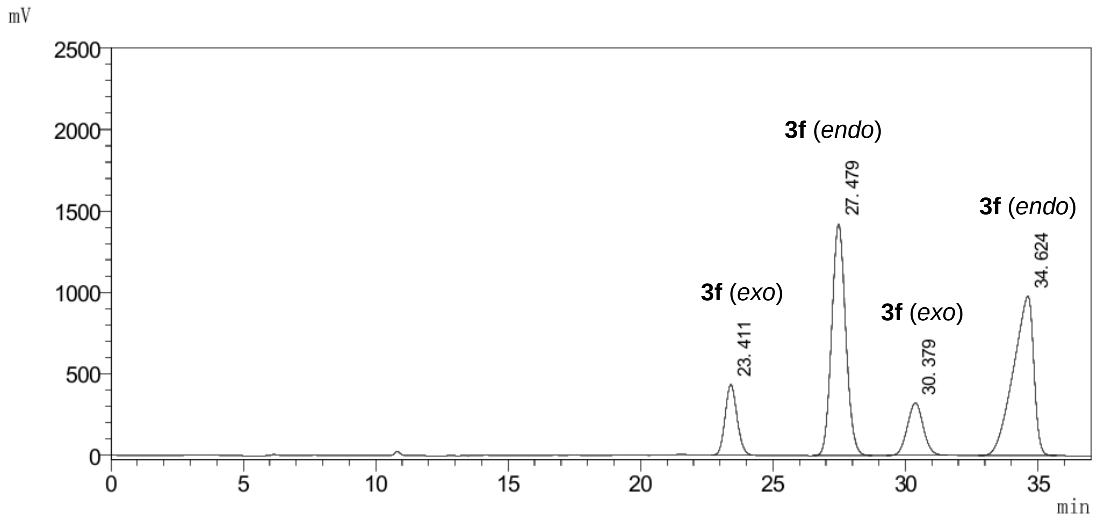

<Peak table>

Peak#	Ret. Time	Area	Height	Conc.	Unit	Mark	Name
1	23.411	13518019	434013	0.000		M	
2	27.479	52426923	1422579	0.000		M	
3	30.379	13449763	322863	0.000		M	
4	34.624	52636351	980594	0.000		M	

b

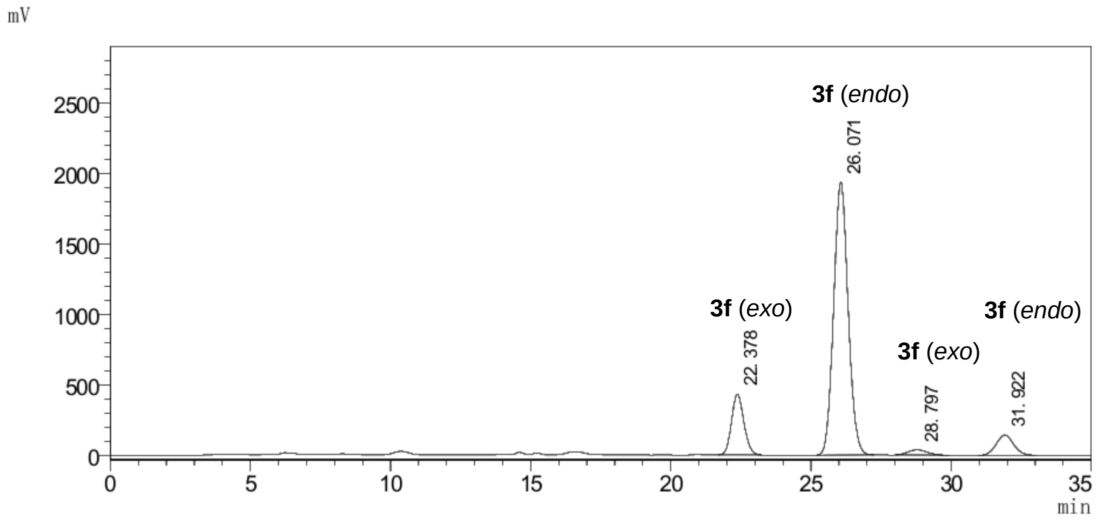

<Peak table>

Peak#	Ret. Time	Area	Height	Conc.	Unit	Mark	Name
1	22.378	13188348	430200	0.000		M	
2	26.071	69324282	1934707	0.000		M	
3	28.797	1778547	36686	0.000		M	
4	31.922	6221126	143456	0.000		M	

Supplementary Fig. 31 a HPLC trace of racemic **3f**. **b** HPLC trace of product **3f** from the Diels-Alder reaction catalyzed by Cu^{2+}·ATP for the *exo* isomer of 76% ee and the *endo* isomer of 84% ee. HPLC condition: Daicel Chiralpak AD column, hexane/*i*-PrOH 90:10, 0.5 mL min^{-1}, 254 nm, 25 °C.

a

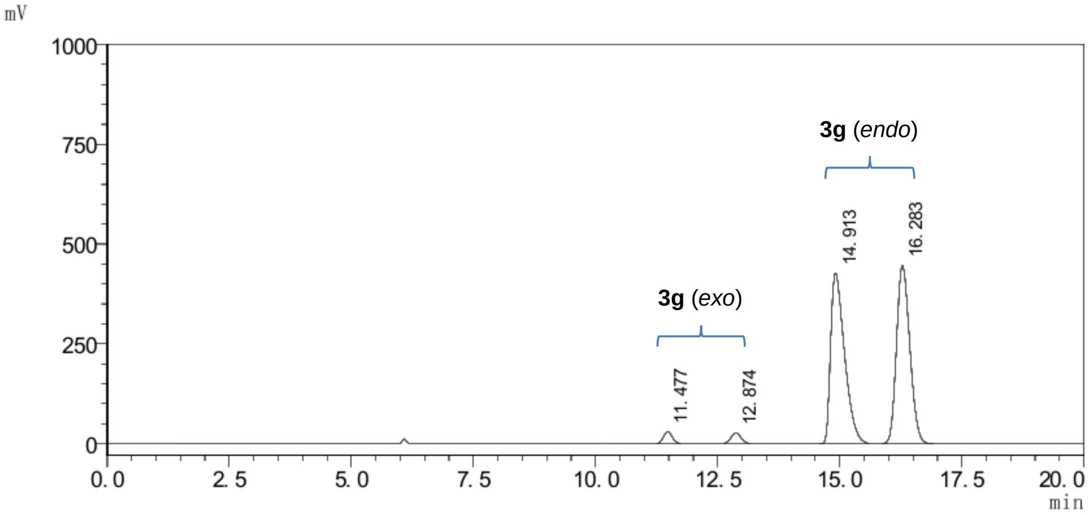

<Peak table>

Peak#	Ret. Time	Area	Height	Conc.	Unit	Mark	Name
1	11.477	364209	29507	0.000		M	
2	12.874	360856	26032	0.000		M	
3	14.913	8462127	427237	0.000		M	
4	16.283	8474746	446715	0.000		M	

b

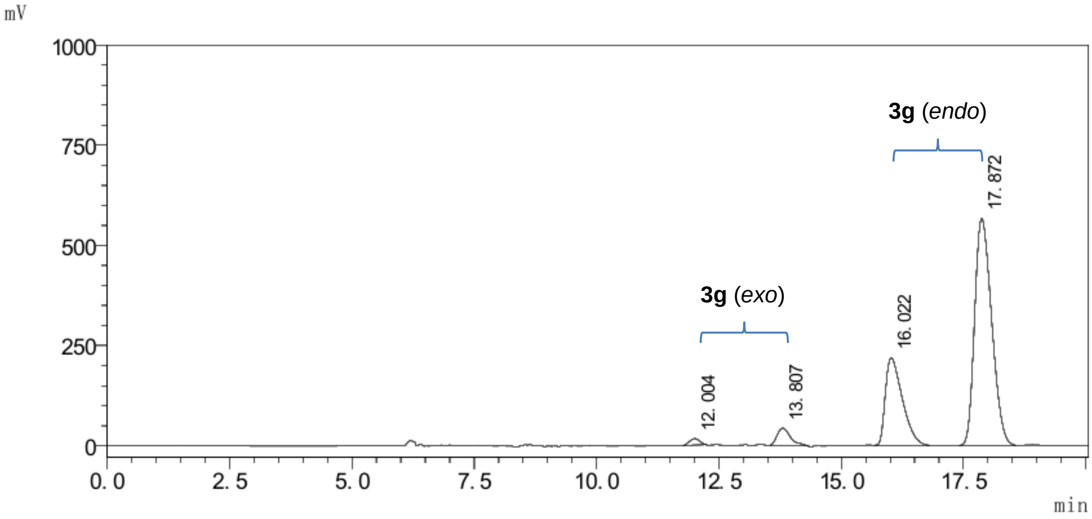

<Peak table>

Peak#	Ret. Time	Area	Height	Conc.	Unit	Mark	Name
1	12.004	237771	16378	0.000		M	
2	13.807	837634	43784	0.000		M	
3	16.022	5279941	218188	0.000		M	
4	17.872	13678379	566640	0.000		M	

Supplementary Fig. 32 a HPLC trace of racemic **3g**. **b** HPLC trace of product **3g** from the Diels-Alder reaction catalyzed by Cu^{2+}·ATP for the *exo* isomer of 56% ee and the *endo* isomer of 44% ee. HPLC condition: Daicel Chiralpak ODH column, hexane/*i*-PrOH 98:2, 0.5 mL min^{-1}, 254 nm, 25 °C.

a

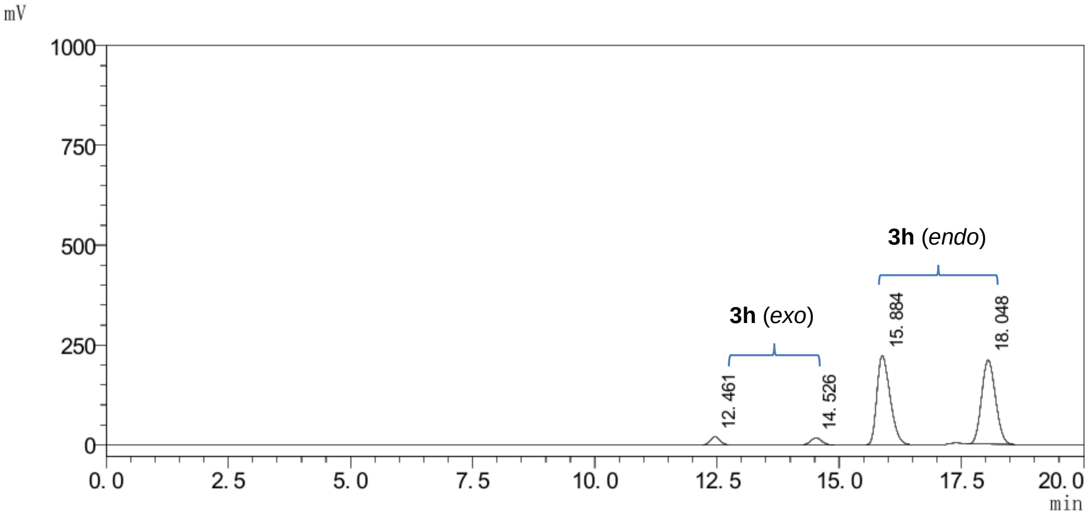

<Peak table>

Peak#	Ret. Time	Area	Height	Conc.	Unit	Mark	Name
1	12. 461	258100	19771	0. 000		M	
2	14. 526	261632	17065	0. 000		M	
3	15. 884	4149071	222725	0. 000		M	
4	18. 048	4169960	211255	0. 000		M	

b

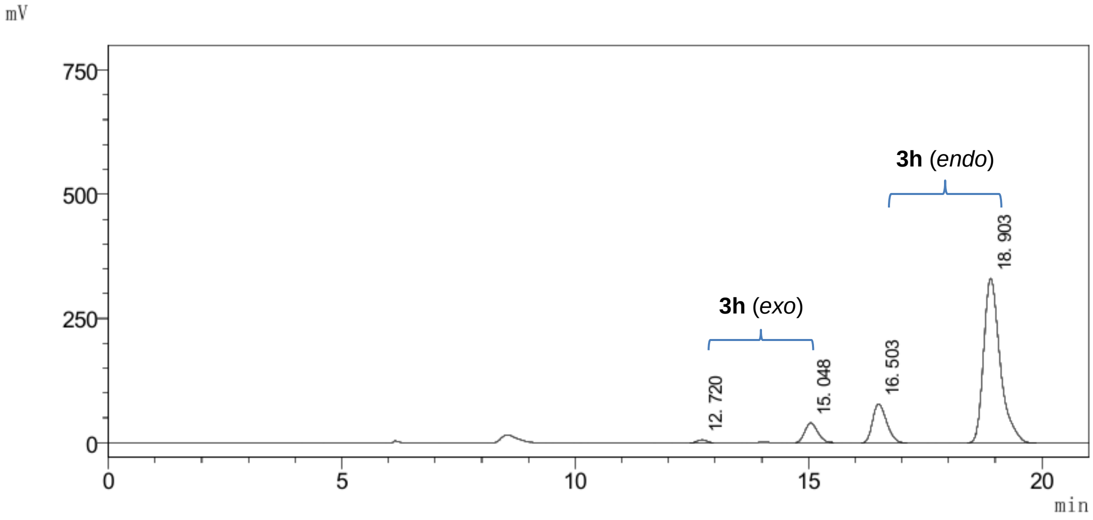

<Peak table>

Peak#	Ret. Time	Area	Height	Conc.	Unit	Mark	Name
1	12. 720	82533	5867	0. 000		M	
2	15. 048	753665	39701	0. 000		M	
3	16. 503	1647025	78644	0. 000		M	
4	18. 903	8191361	331242	0. 000		M	

Supplementary Fig. 33 a HPLC trace of racemic **3h**. **b** HPLC trace of product **3h** from the Diels-Alder reaction catalyzed by Cu^{2+}·ATP for the *exo* isomer of 80% ee and the *endo* isomer of 67% ee. HPLC condition: Daicel Chiralpak ODH column, hexane/*i*-PrOH 98:2, 0.5 mL min^{-1}, 254 nm, 25 °C.

Supplementary References

1. Otto, S., Boccaletti, G. & Engberts, J. A chiral Lewis-acid-catalyzed Diels-Alder reaction. Water-enhanced enantioselectivity. *J. Am. Chem. Soc.* **120**, 4238-4239 (1998).
2. Seelig, B., Keiper, S., Stuhlmann, F. & Jaschke, A. Enantioselective ribozyme catalysis of a bimolecular cycloaddition reaction. *Angew. Chem. Int. Ed.* **39**, 4576-4579 (2000).
3. Roelfes, G. & Feringa, B. L. DNA-based asymmetric catalysis. *Angew. Chem. Int. Ed.* **44**, 3230-3232 (2005).
4. Roelfes, G., Boersma, A. J. & Feringa, B. L. Highly enantioselective DNA-based catalysis. *Chem. Commun.*, 635-637 (2006).
5. Reetz, M. T. & Jiao, N. Copper-phthalocyanine conjugates of serum albumins as enantioselective catalysts in Diels-Alder reactions. *Angew. Chem. Int. Ed.* **45**, 2416-2419 (2006).
6. Coquiere, D., Bos, J., Beld, J. & Roelfes, G. Enantioselective artificial metalloenzymes based on a bovine pancreatic polypeptide scaffold. *Angew. Chem. Int. Ed.* **48**, 5159-5162 (2009).
7. Podtetenieff, J., Taglieber, A., Bill, E., Reijerse, E. J. & Reetz, M. T. An artificial metalloenzyme: creation of a designed copper binding site in a thermostable protein. *Angew. Chem. Int. Ed.* **49**, 5151-5155 (2010).
8. Roe, S., Ritson, D. J., Garner, T., Searle, M. & Moses, J. E. Tuneable DNA-based asymmetric catalysis using a G-quadruplex supramolecular assembly. *Chem. Commun.* **46**, 4309-4311 (2010).
9. Wang, C., *et al.* Enantioselective Diels-Alder reactions with G-quadruplex DNA-based catalysts. *Angew. Chem. Int. Ed.* **51**, 9352-9355 (2012).
10. Bos, J., Fusetti, F., Driessen, A. J. M. & Roelfes, G. Enantioselective artificial metalloenzymes by creation of a novel active site at the protein dimer interface. *Angew. Chem. Int. Ed.* **51**, 7472-7475 (2012).
11. Deuss, P. J., Popa, G., Slawin, A. M. Z., Laan, W. & Kamer, P. C. J. Artificial copper enzymes for asymmetric Diels-Alder reactions. *ChemCatChem* **5**, 1184-1191 (2013).
12. Wilking, M. & Hennecke, U. The influence of G-quadruplex structure on DNA-based asymmetric catalysis using the G-quadruplex-bound cationic porphyrin TMPyP4 center dot Cu. *Org. Biomol. Chem.* **11**, 6940-6945 (2013).
13. Zheng, L., Marcozzi, A., Gerasimov, J. Y. & Herrmann, A. Conformationally constrained cyclic peptides: powerful scaffolds for asymmetric catalysis. *Angew. Chem. Int. Ed.* **53**, 7599-7603 (2014).
14. Zheng, L., *et al.* Turning cucurbit 8 uril into a supramolecular nanoreactor for asymmetric catalysis. *Angew. Chem. Int. Ed.* **54**, 13007-13011 (2015).
15. Filice, M., *et al.* Synthesis of a heterogeneous artificial metallolipase with chimeric catalytic activity. *Chem. Commun.* **51**, 9324-9327 (2015).
16. Li, Y., *et al.* Terpyridine-Cu(II) targeting human telomeric DNA to produce highly stereospecific G-quadruplex DNA metalloenzyme. *Chem. Sci.* **6**, 5578-5585 (2015).
17. Himiyama, T., *et al.* Construction of a hybrid biocatalyst containing a covalently-linked terpyridine metal complex within a cavity of aponitrobindin. *J. Inorg. Biochem.* **158**, 55-61 (2016).
18. Osseili, H., *et al.* Artificial Diels-Alderase based on the transmembrane protein FhuA. *Beilstein J. Org. Chem.* **12**, 1314-1321 (2016).
19. Ghattas, W., *et al.* Artificial metalloenzymes with the neocarzinostatin scaffold: toward a biocatalyst for the Diels-Alder reaction. *ChemBioChem* **17**, 433-440 (2016).
20. Xu, X., *et al.* Enantioselective Diels-Alder reactions using a G-triplex DNA-based catalyst. *Catal. Commun.* **74**, 16-18 (2016).
21. Marek, J. J., Singh, R. P., Heuer, A. & Hennecke, U. Enantioselective catalysis by using short, structurally defined DNA hairpins as scaffold for hybrid catalysts. *Chem. Eur. J.* **23**, 6004-6008 (2017).
22. Himiyama, T., Taniguchi, N., Kato, S., Onoda, A. & Hayashi, T. A pyrene-linked cavity within a beta-barrel protein promotes an asymmetric Diels-Alder reaction. *Angew. Chem. Int. Ed.* **56**, 13618-13622 (2017).
23. Di Meo, T., *et al.* Alpha Rep A3: A versatile artificial scaffold for metalloenzyme design. *Chem. Eur. J.* **23**, 10156-10166 (2017).
24. Marek, J. J. & Hennecke, U. Why DNA is a more effective scaffold than RNA in nucleic acid-based asymmetric catalysis—supramolecular control of cooperative effects. *Chem. Eur. J.* **23**, 6009-6013 (2017).
25. Ghattas, W., *et al.* Receptor-based artificial metalloenzymes on living human cells. *J. Am. Chem. Soc.* **140**, 8756-8762 (2018).
26. Fischer, J., *et al.* Robust and versatile host protein for the design and evaluation of artificial metal centers. *ACS Catal.* **9**, 11371-11380 (2019).
27. Di Meo, T., *et al.* Functionalized artificial bidomain proteins based on an alpha-solenoid protein repeat scaffold: a new class of artificial Diels-Alderases. *ACS Omega* **4**, 4437-4447 (2019).
28. Ghattas, W., *et al.* Cu-II-containing 1-aminocyclopropane carboxylic acid oxidase is an efficient stereospecific Diels-Alderase. *Angew. Chem. Int. Ed.* **58**, 14605-14609 (2019).
29. Qi, Q., *et al.* An efficient cyclic di-AMP based artificial metalloribozyme for enantioselective Diels-Alder reactions. *Eur. J. Org. Chem.* **2020**, 4417-4424 (2020).
30. Otto, S., Bertoncin, F. & Engberts, J. Lewis acid catalysis of a Diels-Alder reaction in water. *J. Am. Chem. Soc.* **118**, 7702-7707 (1996).
31. Wolfe, A., Shimer, G. H., Jr. & Meehan, T. Polycyclic aromatic hydrocarbons physically intercalate into duplex regions of denatured DNA. *Biochemistry* **26**, 6392-6396 (1987).
